# Inflammatory immune profiles associated with disease severity in pulmonary tuberculosis patients with moderate to severe clinical TB or anemia

**DOI:** 10.3389/fimmu.2023.1296501

**Published:** 2023-12-12

**Authors:** Senait Ashenafi, Marco Giulio Loreti, Amsalu Bekele, Getachew Aseffa, Wondwossen Amogne, Endale Kassa, Getachew Aderaye, Susanna Brighenti

**Affiliations:** ^1^ Department of Pathology, School of Medicine, College of Health Sciences, Tikur Anbessa Specialized Hospital and Addis Ababa University, Addis Ababa, Ethiopia; ^2^ Department of Medicine Huddinge, Center for Infectious Medicine (CIM), ANA Futura, Karolinska Institutet, Stockholm, Sweden; ^3^ Department of Internal Medicine, School of Medicine, College of Health Sciences, Tikur Anbessa Specialized Hospital and Addis Ababa University, Addis Ababa, Ethiopia; ^4^ Department of Radiology, School of Medicine, College of Health Sciences, Tikur Anbessa Specialized Hospital and Addis Ababa University, Addis Ababa, Ethiopia

**Keywords:** tuberculosis, inflammation, cytokines, chemokines, disease severity, clinical symptoms, anemia, lung involvement

## Abstract

**Background:**

Immune control of *Mycobacterium tuberculosis* (Mtb) infection is largely influenced by the extensive disease heterogeneity that is typical for tuberculosis (TB). In this study, the peripheral inflammatory immune profile of different sub-groups of pulmonary TB patients was explored based on clinical disease severity, anemia of chronic disease, or the radiological extent of lung disease.

**Methods:**

Plasma samples were obtained from n=107 patients with active pulmonary TB at the time of diagnosis and after start of standard chemotherapy. A composite clinical TB symptoms score, blood hemoglobin status and chest X-ray imaging were used to sub-group TB patients into 1.) mild and moderate-severe clinical TB, 2.) anemic and non-anemic TB, or 3.) limited and extensive lung involvement. Plasma levels of biomarkers associated with inflammation pathways were assessed using a Bio-Plex Magpix 37-multiplex assay. In parallel, Th1/Th2 cytokines were quantified with a 27-multiplex in matched plasma and cell culture supernatants from whole blood stimulated with *M. tuberculosis*-antigens using the QuantiFERON-TB Gold assay.

**Results:**

Clinical TB disease severity correlated with low blood hemoglobin levels and anemia but not with radiological findings in this study cohort. Multiplex protein analyses revealed that distinct clusters of inflammation markers and cytokines separated the different TB disease sub-groups with variable efficacy. Several top-ranked markers overlapped, while other markers were unique with regards to their importance to differentiate the TB disease severity groups. A distinct immune response profile defined by elevated levels of BAFF, LIGHT, sTNF-R1 and 2, IP-10, osteopontin, chitinase-3-like protein 1, and IFNα2 and IL-8, were most effective in separating TB patients with different clinical disease severity and were also promising candidates for treatment monitoring. TB patients with mild disease displayed immune polarization towards mixed Th1/Th2 responses, while pro-inflammatory and B cell stimulating cytokines as well as immunomodulatory mediators predominated in moderate-severe TB disease and anemia of TB.

**Conclusions:**

Our data demonstrated that clinical disease severity in TB is associated with anemia and distinct inflammatory immune profiles. These results contribute to the understanding of immunopathology in pulmonary TB and define top-ranked inflammatory mediators as biomarkers of disease severity and treatment prognosis.

## 1 Introduction

Infection with *Mycobacterium tuberculosis* (Mtb) results in a broad spectrum of clinical presentations and tuberculosis (TB) disease outcomes (1). It is well known that Mtb infection initiates a cascade of both pro-inflammatory and anti-inflammatory mediators in the human host that can both promote and limit bacterial dissemination (2, 3). Consequently, immune control in TB is dictated by a range of soluble factors including cytokines, chemokines, and other inflammatory molecules that can influence disease progression (4). Clinical heterogeneity and disease severity of pulmonary TB are multifactorial but have been described to depend on the extent of clinical TB symptoms including anemia and the level of lung involvement such as fibrosis and cavitation (5). This complexity of TB complicates diagnosis, disease prognosis and treatment decision-making and follow up.

It is well-established that cell-mediated Th1 immunity coordinated by IFN-γ and TNF-α, is required to kill Mtb inside macrophages at the site of infection in the lung (6, 7). Instead, the role of Th2 responses including humoral B cell immunity is more controversial (8) but may be less effective to achieve immune control of TB disease. Th1 cytokines typically activate macrophages and cytolytic T cells to kill intracellular Mtb via induction of reactive oxygen and nitrogen species, antimicrobial peptides, and autophagy (6, 9). Conversely, Th2 cytokines including IL-4, IL-13 and IL-10 promote anti-inflammatory responses that are necessary to prevent pathological inflammation but also reduce the capacity of macrophages and T cells to effectively clear Mtb (10). Instead, Th2 cytokines promote the development of B cells into antibody-producing plasma cells (11) that may inactivate bacteria that are released in the extracellular microenvironment but are likely less operative in Mtb infection control (12). Besides pro-inflammatory cytokines such as IL-6, IL-1β, IL-8, and the classical Th1/Th2 polarization of immune cells, there are many different inflammatory mediators and immunomodulatory proteins that could contribute to immunopathogenic responses in TB. Less explored are for example the IL-20 subfamily of cytokines including IL-19, IL-20, IL-22, and IL-24, that have been shown to contribute to tissue healing processes upon chronic infections and inflammation in the lung by mainly targeting the epithelial barrier at mucosal sites (13). But the IL-20 sub-family have also been shown to down-modulate antigen-specific Th1 and Th17 responses in TB (14). Likewise, IL-11 (15), type I and III interferons (IFN) (16, 17), and acute phase proteins including pentraxin, diverse chemoattractants such as RANTES and MIP-1α and β, or eotaxin (3), as well as proteins that are involved in Mtb-driven tissue remodeling and lung matrix destruction, primarily matrix metalloproteinases (MMPs) (18) or ostepontin (19), have been linked to both protective as well as dysfunctional responses in the regulation of inflammation and development of lung pathology in TB and other diseases.

Recently, we described that anemia of chronic TB disease was associated with more severe clinical disease and elevated levels of pro-inflammatory IL-6 but a suppressed IFN-γ response (20). While it is clear that anemia of chronic diseases such as TB is characterized by systemic inflammation (21, 22), it has not been well described what type of signaling pathways or mediators are involved and how these contribute to immunopathology of Mtb. Now, we extend these findings by assessment of inflammatory mediators and cytokines in different sub-groups of pulmonary TB patients based on clinical disease severity, anemia or radiological findings. Plasma levels of biomarkers associated with inflammation pathways including pro-inflammatory and the IL-10 family of cytokines, type I/II/III IFN, TNF ligands and receptors as well as Th1/Th2 and immunomodulatory cytokines and proteins involved in tissue-remodeling and wound-healing were assessed using 37- and 27-multiplex assays, respectively. While these results add to the pipeline of studies using a proteomics approach to improve TB diagnosis and prognosis, we anticipate that these data will also contribute to the understanding of pathophysiology in TB by assessing the inflammatory profile in different TB patient subgroups.

## 2 Materials and methods

### 2.1 Study cohort

HIV-negative patients >18 years, with newly diagnosed active pulmonary TB (n=107) and healthy controls (n=19) were recruited at the Chest Unit, Department of Internal Medicine, Tikur Anbessa Specialized University Hospital in Addis Ababa, Ethiopia after written and signed informed consent. The study subjects were enrolled in a previously conducted clinical trial (23) that was approved by the ethical review boards in Ethiopia and Sweden, and registered at www.clinicaltrials.gov, NCT01698476, prior to inclusion of the first patient. Secondary endpoint measures including multiplex protein assays were conducted on one third (107/345; 31%) of the enrolled TB patients in the intervention (n=53) and placebo (n=54) treatment groups, respectively. A confirmed TB diagnosis was based on a positive sputum microscopy or Mtb-culture, and/or clinical symptoms and chest X-ray findings consistent with TB, i.e., clinical TB defined according to WHO criteria. HIV-infection, multidrug-resistant TB (MDR-TB) or extrapulmonary TB, anti-TB treatment in the past 2 years, pregnant women, patients with kidney or liver disease, cancer or autoimmune diseases were excluded from the study. Healthy controls (n=19) were also included to assess baseline levels of inflammatory mediators.

### 2.2 Clinical measurements

A previously validated clinical TB score (23–25) was used to assess clinical disease severity. This is a numerical composite TB score (2-point scale: symptom absent (0p) or present (1p), max 13p) that included self-reported clinical symptoms (cough, night sweats, and chest pain), as well as different variables documented upon clinical examination including conjunctival pallor, hemoptysis, dyspnea, tachycardia, positive findings at lung auscultation, fever, low BMI (<18 and/or <16), and low mid-upper arm circumference (MUAC) (<22 and/or <20 cm). Weight and height were measured to determine BMI (weight/(height^2^)) while a measuring tape was used to assess MUAC. BMI was defined as underweight ≤ 18.5 Kg/m^2^ or normal weight > 18.5 Kg/m^2^), while MUAC was defined as underweight ≤ 21cm or normal weight > 21cm. Patients were sub-grouped into mild (TB score 0-6) or moderate-severe (TB score 7-13) clinical TB disease based on the average TB score obtained among n=107 included TB patients (**Table 1**). In addition to the TB score, patients were sub-grouped in anemic and non-anemic TB disease based on the normal reference blood Hb values for males (<13.5 g/dl) and females (<12 g/dl) (26).

**Table 1:** Baseline characteristics of study subjects.

	Human inflammation 37-plex		Human cytokine 27-plex	
Variables^a^	Pulmonary TB (n=107)	Controls (n=12)	Pulmonary TB (n=60)	Controls (n=19)
Age in years (range)	25 (18-63)	30 (18-57)	24.5 (18-70)	29 (18-57)
Gender (M/F)	58/49	4/8	30/30	5/14
Sputum-smear pos/neg (no)	94/13	nd	54/6	nd
TB score (0-13p)	6	nd	6	nd
Mild/Mod-sev TB (no)^b^	58/49	nd	28/32	nd
Hemoglobin (g/dL) (M/F)	12.7 (13/12.4)	nd	13 (13/12.6)	nd
Non-anemic/Anemic TB (no)^c^	50/57	nd	28/32	nd
Chest X-ray grade (0-3p)^d^	3	nd	3	nd
Limited/extensive involvement (no)^e^	41/59	nd	25/29	nd
BMI (kg/m2)	17.75	21.7****	17.8	22.6****
Underweight ≤18.5/Normal weight >18.5	76/31	0/11	42/18	2/16
MUAC (cm)	21.5	25.75****	21.5	27****
ESR (mm/hour)	50	nd	50	nd
WBC counts (10^9^ cells/L)	7.8	nd	7.6	nd
CD4 T cell counts (cells/mm^3^)	419.5	665.5***	397.5	631***
CD8 T cell counts (cells/mm^3^)	327.5	403	300	407*
QuantiFERON (IU/ml)^f^	2.71	1.29	2.79*	0.71

a Data are presented as numbers (no) or median.

b According to numerical TB score (0–6 p = mild TB, >6 = moderate-severe TB).

c According to normal blood Hb reference value for males (<13.5 g/dL) and females (<12 g/dL). Blood Hb was not determined for the healthy controls.

d According to radiological chest X-ray grading (0-2 = limited lung involvement, 3 = extensive lung involvement).

e n=7 patients did not have chest X-ray data.

f Determined using the QuantiFERON TB Gold In-Tube (QFTG) assay.

Statistical difference between TB patients and healthy controls was determined using a Mann-Whitney U test, except for blood Hb comparing the difference between males and females. *p≤0.05, ***p≤0.001, and ****p≤0.0001.

mod-sev TB, moderate-severe TB; BMI, body mass index; MUAC, mid-upper arm circumference; WBC, white blood cell count; ESR, erythrocyte sedimentation rate; nd, not determined.

An experienced radiologist examined standard full-size posteroanterior chest X-rays and graded pathological lung involvement at the time of diagnosis using the diagnostic standards and classifications of TB described by the American Thoracic Society and as previously reported (23, 27). Pulmonary pathologies included infiltrates, consolidations or opacifications, lesions such as nodules and granulomas, fibrosis, and cavitation. Radiological findings were graded as normal (grade 0): no lung involvement; mild/minimal (grade 1): non-confluent uni- or bilateral lung involvement confined to the apical segment with no visible cavitation; moderately advanced (grade 2): disseminated uni- or bilateral lung involvement in the absence or presence of cavitation (cavity size < 4 cm); or far advanced (grade 3): disseminated uni- or bilateral lung involvement with cavitation (cavity size > 4 cm). For mild TB, the extent of lung involvement did not exceed the volume of the lung on one side above the second costochondral junction or the 4th or 5th vertebrae. For moderately and far advanced TB, the extent of lung involvement comprised disseminated lesions of slight to moderate density that covered the total volume of one lung or equivalent volumes in both lungs, or dense and confluent lesion(s) that were limited to one third of the volume of one lung lobe. The study cohort of n=100 subjects (no available chest X-ray data from n=7) was sub-grouped into patients with more limited (grade 0-2) or extensive (grade 3) lung involvement.

### 2.3 Laboratory measurements

Peripheral blood was obtained at the time of inclusion and at week 4, 8 and 16 after start of standard chemotherapy from a sub-group of subjects including equal numbers of patients with mild (n=36), moderate (n=36), or severe (n=35) TB disease, determined using a composite clinical TB score described above (24). Blood analyses and chest X-ray were used to further sub-group TB patients into anemic and non-anemic patients or extensive and limited lung involvement using Hb values and imaging data, respectively. Whole blood samples were used for blood chemistry analyses and for Mtb-specific restimulation of blood cells *in vitro* using QuantiFERON-TB Gold in-Tube (Cellestis; Statens Serum Institut, Denmark), according to the manufacturer´s instructions. Plasma samples were isolated from Lymphoprep (Alere technologies, Norway) centrifugation (2000 rpm, 20-30 min at room temperature) of blood in Leucosep tubes (Greiner Bio-One, Austria). Plasma aliquots were stored at -80°C until multiplex analyses. Blood hemoglobin, white blood cell count (WBC) (Abbott, Il, USA), erythrocyte sedimentation rate (ESR), CD4 and CD8 T cell counts (BD Biosciences, NJ, USA) were assessed at the International Clinical Laboratory (ICL) in Addis Ababa, Ethiopia.

### 2.4 Multiplex assays

Plasma levels of inflammation-associated mediators were assessed using a Bio-Plex Pro Human Inflammation 37-Plex Panel 1, in 96-well plate format for the detection of 37 inflammation biomarkers (171AL001M; Bio-Rad, Hercules, CA). In parallel, secreted proteins in plasma and matched cell culture supernatants (n=79) obtained from whole blood stimulated with Mtb-antigens using the QuantiFERON-TB test, were quantified using a Bio-Plex Pro Human Cytokine 27-Plex Panel, in 96-well plate format for the detection of 27 Th1/Th2 cytokines and chemokines (M500KCAF0Y, Bio-Rad, Hercules, CA). Samples were analyzed using a Bio-Plex^®^ MAGPIX™ Multiplex Reader and the Bio-Plex Manager software (Bio-Rad, Hercules, CA).

Blood samples can be used to assess both cells and soluble factors that leak into the peripheral circulation from the site of infection. Inflammatory mediators are produced by diverse cell types including both innate and adaptive immune cells as well as epithelial cells, while specific Th1/Th2 cytokines are mostly produced by activated T cells. In a small pilot experiment, the inflammatory markers in the 37-plex were readily detectable in ex vivo collected plasma samples, while cytokines and chemokines in the 27-plex were expressed at very low levels. To enhance the detection levels of the 27-plex markers, the QuantiFERON-TB assay was used to re-stimulate Mtb-specific T cells in whole blood samples to promote accumulation of cytokines in cell culture supernatants.

### 2.5 Statistical analysis

Unsupervised analyses of acquired 37-plex and 27-plex data were performed in R programming language (R) version 4.2.2 (R Core Team, 2022) within the RStudio integrated development environment version 2023.03.1 + 446. These methods included random forest (RF) for classification and ranking of the most important mediators and principal component analysis (PCA) for dimensionality reduction and clusters analyses. Principal component 1 (PC1) and 2 (PC2), were presented in box plot graphs showing interquartile range and median. The normality of the data was tested using Shapiro–Wilk normality test. A non-parametric Mann-Whitney test or Kruskal-Wallis and Dunn’s post-test as well as Holm’s multiple correction test was used for comparison of two or multiple unpaired groups. A p-value < 0.05 was considered significant. Statistical analysis and box plot graphs were generated via the package ‘ggstatsplot’ (28).

Manual analyses of data were performed in GraphPad Prism 9.0, using an unpaired t-test for normally distributed data, while data that did not pass a normality test was analyzed using the Mann–Whitney test. Box and whiskers plots show data as median and 10-90 percentile, while violin plots shown median and range. Bar graphs show mean and 95% confidence interval (CI) or standard error (SE). Correlation was determined using Spearman´s correlation test. A repeated measurements ANOVA and Sidak post-test was used for comparison of longitudinal data presented in bar graphs as mean and standard error at week 0, 4, 8 and 16. A p-value < 0.05 was considered significant.

## 3 Results

### 3.1 Clinical and laboratory characteristics of TB patient sub-groups

Baseline data from TB patients and controls is presented in **Table 1**. Enrolled TB patients were young, median 25 years, and around 70% of the TB subjects were underweight and accordingly both BMI and MUAC were significantly lower (p<0.0001) in the TB patients compared to healthy controls. Peripheral CD4 T cell counts have been shown to be associated with disease severity in HIV-negative TB patients (29) and were also significantly reduced (p<0.001) in the TB patients compared to the controls. In the subjects used in the 27-plex assay, CD8 T cell counts were higher (p<0.05) in the controls while IFN-γ levels detected by the QuantiFERON test were significantly higher (p<0.05) in the TB patients.

**Figure 1:**
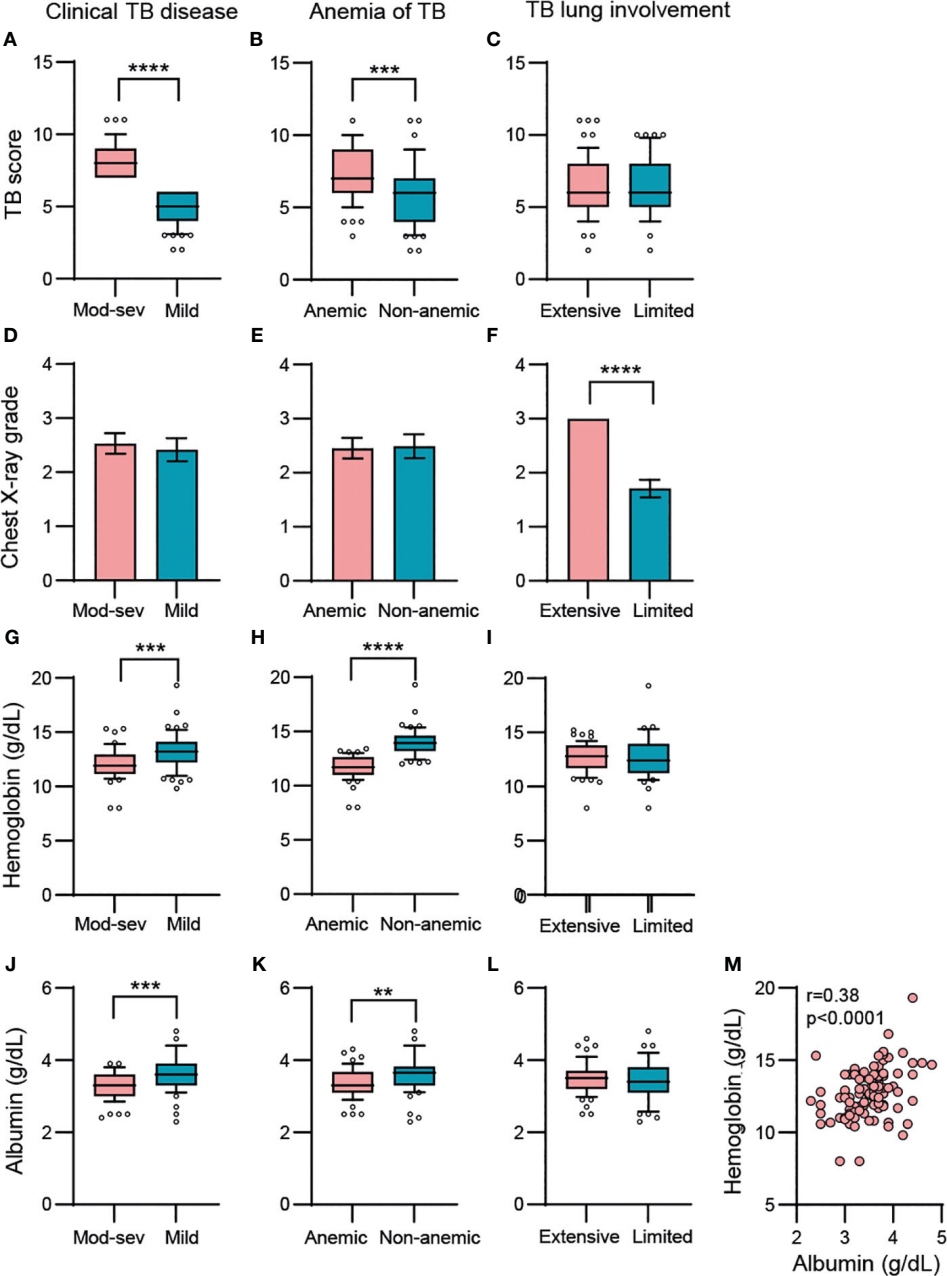
Baseline variables in clinical TB disease (mod-sev vs mild TB), anemia of TB (anemic vs non-anemic TB) and TB lung involvement (extensive vs limited TB). **(A-C)** Clinical TB score, **(D-F)** chest X-ray grade, **(G-I)** blood Hb, **(J-L)** serum albumin levels. **(M)** Correlation analysis between blood Hb and serum albumin including all TB patients was determined using Spearman´s correlation test. Data is presented in box and whiskers plots (median and 10-90 percentile) or bar graphs (mean and 95% CI) and was analyzed using a Mann-Whitney *U* test or an unpaired t-test. **p≤0.01, ***p≤0.001, and ****p≤0.0001.

Chronic inflammation was mapped in four sub-groups of study subjects including 1.) pulmonary TB patients (n=107) vs healthy controls (HC) (n=12/19), 2.) patients with mild (n=58) vs moderate-severe (mod-sev) (n=49) clinical TB, 3.) anemic (n=57) vs non-anemic (n=50) patients and 4.) patients with limited (n=41) vs extensive (n=59) lung involvement. The proportion of cavitary TB in the cohort was high, around 84% (data not shown). As expected, the composite clinical TB score was significantly higher in patients with moderate/severe TB (p<0.0001) and in patients with anemia (p=0.0003) (**Figures 1A, B**). There was no difference in the TB score comparing extensive versus limited TB disease (**Figure 1C**), despite a higher radiological score in TB patients with extensive lung involvement (**Figure 1F**). Contrary, chest X-ray grading was similar in mild versus mod-sev TB and anemic versus non-anemic TB (**Figures 1D, E**). Blood Hb was naturally lower in anemic TB patients (p<0.0001) but also in patients with mod-sev TB (p=0.0001) (**Figures 1G, H**). As an indicator of inflammation severity (30), serum albumin levels were found to be significantly lower in mod-sev TB (p=0.0007) and anemic TB patients (p=0.001) (**Figures 1J, K**), and there was a positive correlation (r=0.38; p<0.0001) between blood Hb and serum albumin levels in all TB patients (**Figure 1M**). Serum albumin was similar in TB patients with extensive and limited chest X-ray findings (**Figure 1L**).

### 3.2 Longitudinal changes of soluble mediators demonstrate a normalization of inflammation in response to anti-TB treatment

The protein concentrations in plasma for the 37-plex and in plasma and QuantiFERON supernatants for the 27-plex are summarized in **Supplementary Tables 1**, **2A, B, 3**, respectively. All marker abbreviations are listed in a footnote (**Table S1**, **S2A, B**). First, unsupervised analysis was exploited to provide an unbiased view of the inflammatory immune profile in the different study sub-groups. Random forest (RF) analyses ranked the importance of the markers in each multiplex assay to better discriminate between the different study sub-groups. Principal component analyses (PCA) were used to reduce data dimensionality when comparing different TB disease groups and to enable clusters generation. It was apparent that the magnitude of the detected 27-plex cytokine responses was clearly lower in plasma with many data points below the detection levels. Therefore, data obtained with restimulated whole blood samples were used for the unsupervised analyses.

**Figure 2:**
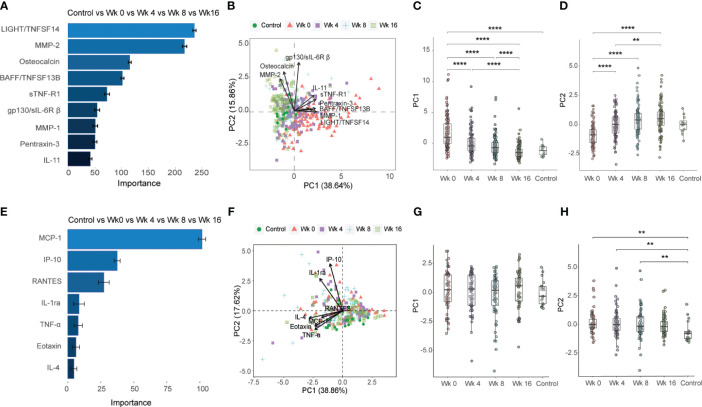
Importance ranking and dimensionality reduction facilitate cluster generation of multiplex data from longitudinal samples obtained from TB patients and healthy controls. **(A)** Random forest (RF) analyses of acquired 37-plex inflammation panel obtained at week 0, 4, 8 and 16 from TB patients and healthy controls. **(B)** Principal component analysis (PCA) showing the longitudinal response in all TB patients and controls including top-9 inflammation markers ranked by RF analyses. Scatter box plots of **(C)** PC1 and **(D)** PC2. **(E)** RF of acquired 27-plex cytokine panel obtained at week 0, 4, 8 and 16 from TB patients and healthy controls. **(F)** PCA showing the longitudinal response all TB patients and controls including top-7 cytokines ranked by RF analyses. Scatter box plots of PC1 **(G)** and PC2 **(H)**. Multiplex data in **(A-D)** were obtained from plasma samples, while multiplex data in **(E-H)** were obtained from QuantiFERON supernatants from whole blood samples. Data is presented as mean and standard error and all principal components were assessed using Kruskal-Wallis and Dunn´s post-test with multiple comparisons corrected by Holm–Bonferroni method. **p≤0.01, ***p≤0.001, and ****p≤0.0001.

In the first part of the analyses, baseline (week 0) and longitudinal treatment responses (week 4, 8 and 16) detected in the TB patients were plotted together with the control group (**Figures 2A-H**). For the 37-plex and 27-plex, 9 inflammation markers and 7 cytokines were ranked according to the order of importance based on a minimum of 50% variance in the PCA analyses (**Figures 2A, B, E, F**, **Supplementary Figure 1**). For the 37-plex, these included the TNF superfamily members (TNFSF) LIGHT/TNFSF14 and B cell activating factor (BAFF)/TNFSF13B, MMP-1 and 2, Osteocalcin, soluble TNF receptor (sTNF-R) 1 as well as sIL-6R-β (gp130), pentraxin-3, and the IL-6 family member IL-11 (**Figures 2A, B**). While these top-ranked 9 inflammation markers created diverse clusters at week 0, 4, and 8, the week 16 clusters overlapped to a great extent with the healthy controls (**Figure 2B**), which confirmed that the majority of the inflammatory markers (PC1: 38.64%) were normalized upon successful anti-TB treatment (week 0 vs week 16: p<0.0001) (**Figure 2C**). In addition, some markers (PC2: 15.86%) differed significantly comparing baseline data to week 4, 8 and 16 (p<0.01 – <0.0001), but this variability was gradually reduced from week 0 to week 16 (p<0.0001) (**Figure 2D**).

The 7 top-ranked markers in the corresponding 27-plex analyses were mostly chemokines including monocyte chemoattractant protein (MCP-1), IP-10 (CXCL10), RANTES (CCL5), IL-1 receptor antagonist (RA), TNF-α, eotaxin and IL-4 (**Figures 2E, F**). However, these markers did not discriminate the baseline and different follow up time-points to the same extent as the 37-plex data (**Figure 2F**). The PC1 (38.86%) analyses showed that the cluster of ranked cytokines did not differ from baseline to week 16 or compared to the control group (**Figure 2G**), while the PC2 (17.62%) variance between week 0, 4 or 8 and the controls was significant (week 0 vs HC: p=0.002, week 4 vs HC: p=0.007; week 8 vs HC: p=0.01) (**Figure 2H**).

### 3.3 Distinct sets of inflammation markers separate different TB disease severity groups

**Figure 3:**
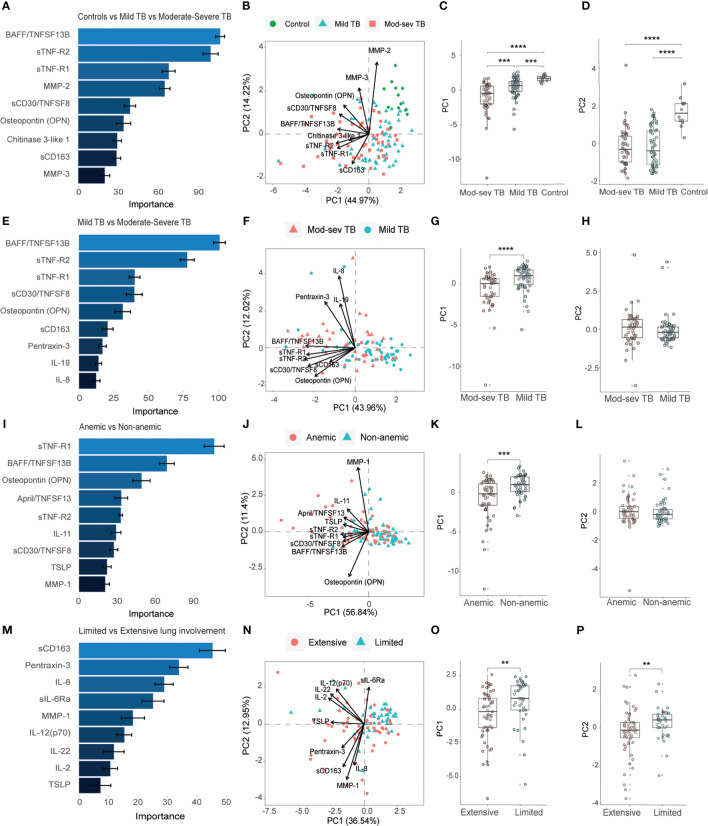
Importance ranking and dimensionality reduction facilitate clusters generation of multiplex inflammation data from different sub-groups of TB patients and healthy controls. **(A)** Random forest (RF) analyses of acquired 37-plex inflammation panel from patients with mild and mod-sev TB disease and healthy controls. **(B)** Principal component analysis (PCA) showing the baseline response in patients with mod-sev and mild TB as well as controls including top-9 inflammation markers ranked by RF analyses. Scatter box plots of **(C)** PC1 and **(D)** PC2. **(E)** RF analyses of acquired 37-plex inflammation panel from patients with mild and mod-sev TB disease. **(F)** PCA showing the baseline response in patients with mod-sev and mild TB including top-9 inflammation markers ranked by RF analyses. Scatter box plots of **(G)** PC1 and **(H)** PC2. **(I)** RF analyses of acquired 37-plex inflammation panel from patients with and without anemic TB disease. **(J)** PCA showing the baseline response in patients with mod-sev and mild TB including top-9 inflammation markers ranked by RF analyses. Scatter box plots of **(K)** PC1 and **(L)** PC2. **(M)** RF analyses of acquired 37-plex inflammation panel from TB patients with limited or extensive lung involvement. **(N)** PCA showing the baseline response in patients with extensive and limited lung involvement including top-9 inflammation markers ranked by RF analyses. Scatter box plots of **(O)** PC1 and **(P)** PC2. All multiplex data were obtained from plasma samples. Data is presented as mean and standard error and all principal components were assessed using Kruskal-Wallis and Dunn´s post-test with multiple comparisons corrected by Holm–Bonferroni method or Mann-Whitney *U* test. **p≤0.01, ***p≤0.001, and ****p≤0.0001.

Next, the inflammatory response at baseline in TB patients with mild or mod-sev disease was analyzed together with (**Figures 3A-D**) and without (**Figures 3E-H**) the healthy control group. When the control group was included, the markers with highest discriminatory importance included BAFF, sTNF-R1 and 2, MMP-2, sCD30, Osteopontin (or ETA-1, early T lymphocyte activation), Chitinase-3-like protein 1 (CHI3L1), sCD163 and MMP-3 (**Figures 3A, B**). Compared to the longitudinal treatment analyses for all TB patients (**Figures 2A, B**), only three markers overlapped with baseline analyses of clinical TB disease severity, including BAFF, sTNF-R1 and MMP2 (**Figures 3A, B**) that were all ranked to be of high importance in the respective RF analyses. For clinical TB disease severity, PC1 (44.97%) analyses demonstrated a significant difference between mod-sev TB versus mild TB (p=0.0003) and compared to the healthy control (mod-sev TB vs HC, p<0.0001; mild TB vs HC, p=0.0009) (**Figure 3C**). There was also a difference in PC2 (14.22%) comparing the clinical disease groups to controls (mod-sev TB or mild TB vs HC, p=0.000008), but no difference comparing mod-sev and mild TB (**Figure 3D**). Six of the top-ranked 9 markers remained listed in the RF plot when mild TB was compared to mod-sev TB in the absence of the controls (**Figure 3E**). Instead of MMP-2, 3, and CHI3L1, the acute phase protein, pentraxin-3, was ranked together with IL-19 and IL-8 (**Figures 3E, F**). PC1 (43.96%) analysis confirmed significant differences in mod-sev compared to mild TB (p=0.00005), while no difference in PC2 (12.02%) was detected (**Figures 3G, H**).

Thereafter, we continued to explore the inflammatory baseline profile comparing anemic to non-anemic TB patients (**Figures 3I-L**) as well as patients with extensive compared to limited lung involvement (**Figures 3M-P**). Five of the top-ranked 9 markers comparing mod-sev to mild TB (**Figures 3E, F**) were also listed comparing anemic to non-anemic TB patients, while 4 markers were unique, a proliferation-inducing ligand (APRIL/TNFSF13), IL-11, thymic stromal lymphopoietin (TSLP) and MMP-1, in discrimination of anemic and non-anemic TB patients (**Figures 3I, J**). Like clinical TB disease severity, PC1 (56.84%) analysis demonstrated significant differences comparing anemic to non-anemic TB patients (p=0.0002), with no difference in PC2 (11.40%) variance (**Figures 3K, L**). Contrary, three (sCD163, pentraxin-3 and IL-8) of the top-ranked 9 markers for clinical disease severity (**Figures 3E, F**) overlapped with the RF analyses comparing extensive with limited lung disease (**Figures 3M, N**), suggesting that there may be differences in inflammatory mediators involved in clinical and radiological disease severity. The variance in both PC1 (36.54%) and PC2 (12.95%), were significantly different in patients with extensive compared to limited lung disease (p=0.002 and p=0.005, respectively) (**Figures 3O, P**).

### 3.4 Diverse cytokine and chemokines profile separate different TB disease severity groups

**Figure 4:**
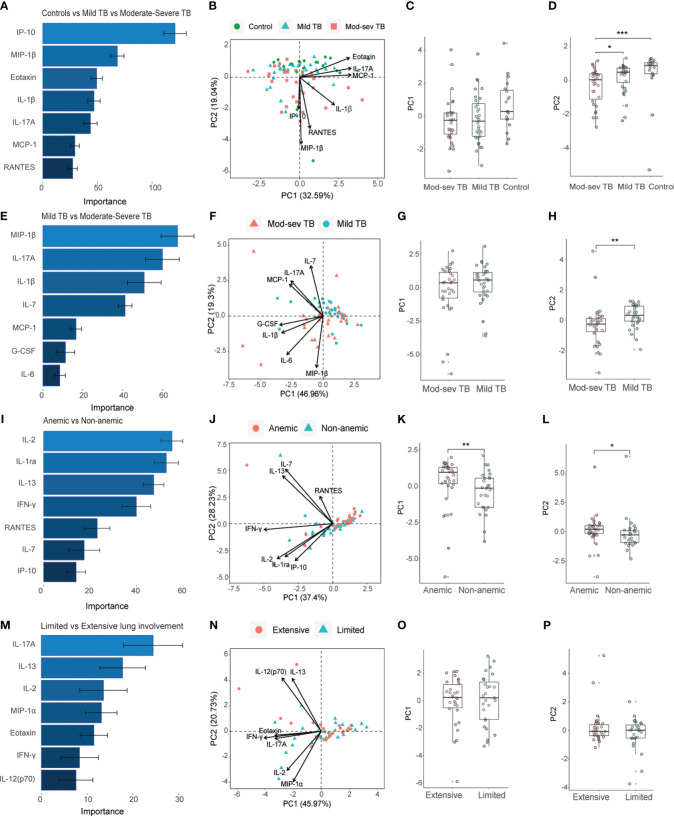
Importance ranking and dimensionality reduction facilitate clusters generation of multiplex cytokine data from different sub-groups of TB patients and healthy controls. **(A)** Random forest (RF) analyses of acquired 27-plex cytokine panel from patients with mild and mod-sev TB disease and healthy controls. **(B)** Principal component analysis (PCA) showing the baseline response in patients with mod-sev and mild TB as well as controls including top-7 cytokines ranked by RF analyses. Scatter box plots of **(C)** PC1 and **(D)** PC2. **(E)** RF analyses of acquired 27-plex cytokine panel from patients with mild and mod-sev TB disease. **(F)** PCA showing the baseline response in patients with mod-sev and mild TB including top-7 cytokines ranked by RF analyses. Scatter box plots of **(G)** PC1 and **(H)** PC2. **(I)** RF analyses of acquired 27-plex cytokine panel from patients with and without anemic TB disease. **(J)** PCA showing the baseline response in patients with mod-sev and mild TB including top-7 cytokines ranked by RF analyses. Scatter box plots of **(K)** PC1 and **(L)** PC2. **(M)** RF analyses of acquired 27-plex cytokine panel from TB patients with limited or extensive lung involvement. **(N)** PCA showing the baseline response in patients with extensive and limited lung involvement including top-7 cytokines ranked by RF analyses. Scatter box plots of **(O)** PC1 and **(P)** PC2. All multiplex data were obtained from QuantiFERON supernatants from whole blood samples. Data is presented as mean and standard error and all principal components were assessed using Kruskal-Wallis and Dunn´s post-test with multiple comparisons corrected by Holm–Bonferroni method or Mann-Whitney *U* test. *p≤0.05, **p≤0.01, and ***p≤0.001.

The baseline inflammatory response was further explored using the Th1/Th2 cytokine 27-plex in patients with mod-sev and mild clinical TB in the presence (**Figures 4A-D**) or absence (**Figures 4E-H**) of the healthy control group. Four out of 7 markers overlapped among the top-ranked cytokines used to discriminate mod-sev TB from mild TB in the presence or absence of the controls (**Figures 4A–H**). While macrophage inflammatory protein (MIP-1β or CCL4), IL-17A and IL-1β were classified to be of high importance to separate the different groups, IFN-γ-induced protein 10 (IP-10 or CXCL10) and eotaxin were more related to separation of TB patients and controls (**Figures 4A, B**), whereas IL-7 was ranked of high importance to discriminate between mod-sev and mild TB (**Figures 4E, F**). PCA showed a low discriminatory power of PC1 (32.59%), while PC2 (19.04%) analysis showed that mod-sev TB was significantly different compared to both mild TB (p=0.04) and the healthy control (p=0.0003) (**Figures 4C, D**). Exclusion of the controls did not alter the relationship notably, but there was no difference between the disease severity groups detected for PC1 (46.96%) but significant differences (p=0.002) comparing these groups in PC2 (19.30%) (**Figures 4G, H**).

Comparison of anemic to non-anemic TB patients (**Figures 4I-L**) disclosed only one overlapping cytokine, IL-7, that was ranked as a top-classifier to separate both mod-sev from mild TB and anemic from non-anemic TB disease. Other markers ranked to separate anemia from non-anemia included IL-2, IL-1 receptor antagonist (RA), IL-13, IFN-γ, RANTES (CCL5) and IP-10 (**Figures 4I, J**). Based on these cytokines and chemokines, the PC1 (37.4%) and PC2 (26.3%) analyses revealed significant discrepancies between the anemic and non-anemic TB groups (p=0.008 and p=0.02, respectively) (**Figures 4K, L**). Finally, the top-7 ranked markers to separate extensive from limited lung involvement partly overlapped with the set of cytokines determined to separate mod-sev and mild TB (IL-17A, eotaxin) as well as anemic and non-anemic TB patients (IL-2, IL-13, IFNγ), while unique cytokines included MIP-1α (CCL3) and IL-12 (**Figures 4M, N**). However, this cytokine module did not separate extensive from limited lung disease in either PC1 (45.97%) or PC2 (20.73%) (**Figures 4O, P**).

### 3.5 Inflammation profiles that associate with TB disease severity or immune control

**Figure 5:**
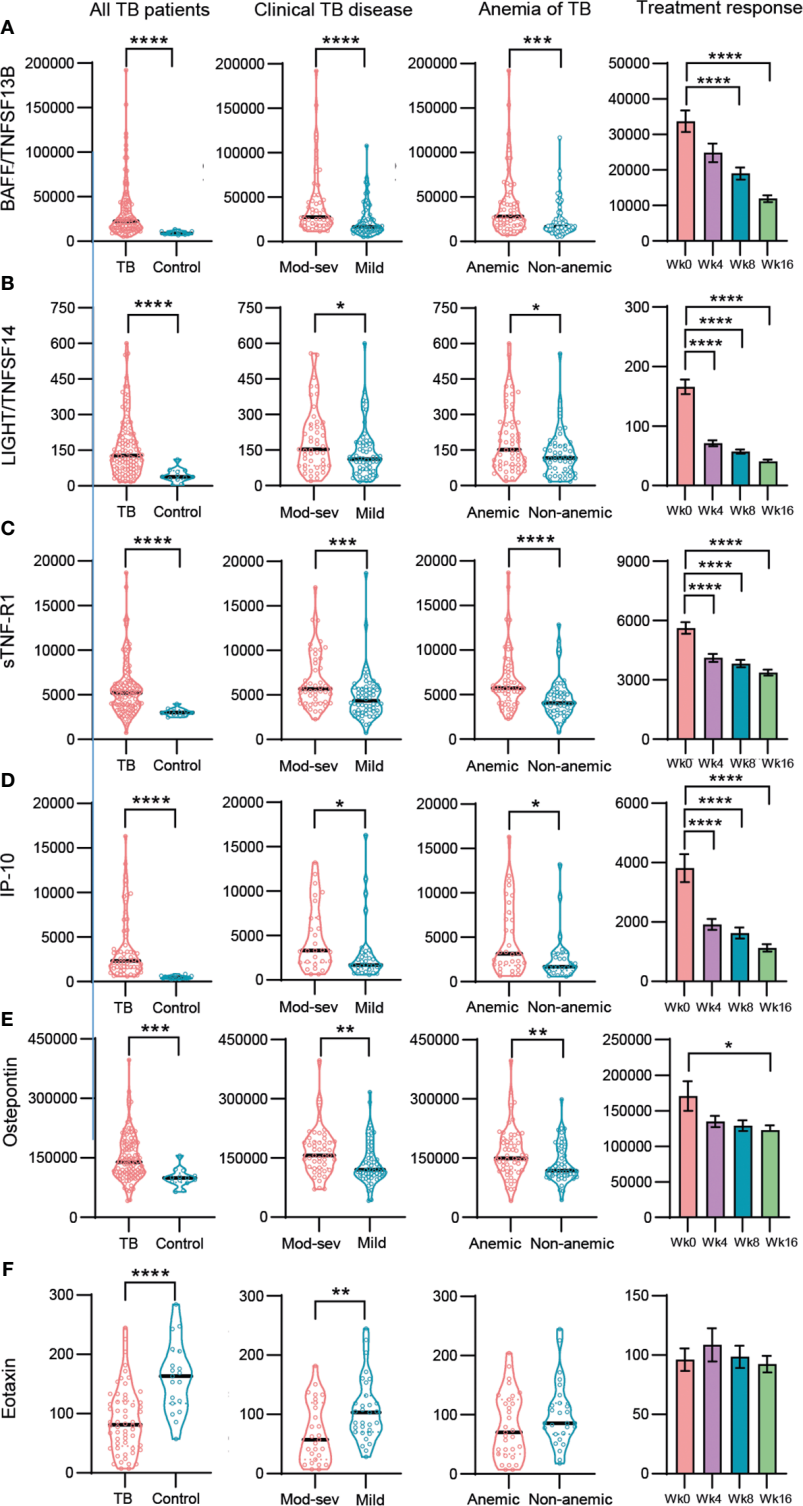
Baseline and longitudinal responses of selected top-ranked inflammation mediators and cytokines in different sub-groups of TB patients and healthy controls. **(A)** BAFF/TNFSF13B, **(B)** LIGHT/TNFSF14, **(C)** sTNF-R-1, **(D)** IP-10, **(E)** Osteopontin, **(F)** Eotaxin. Biomarker levels (pg/ml) is shown in all TB patients (TB patients vs controls), clinical TB disease (mod-sev vs mild TB), anemia of TB (anemic vs non-anemic TB) and treatment response (week 0, 4, 8 and 16). Data is presented in violin plots (median and range) or bar graphs (mean and SE) and was analyzed using a Mann-Whitney *U* test or a repeated measurements ANOVA and Sidak post-test. *p≤0.05, **p≤0.01, ***p≤0.001, and ****p≤0.0001.

To explore possible findings that were not uncovered by the unsupervised methods, manual analysis of the multiplex data was performed in more detail. About 25% of the inflammation markers and cytokines in the multiplex assays tested were significantly higher in TB disease compared to the healthy controls, while only five mediators, the type IV collagenases, MMP2 and 3, the bone matrix protein, osteocalcin, and the chemokines, eotaxin and MCP-1, were higher in the controls (**Table S1**, **S2A, B**). Most, but not all markers upregulated in TB patients were also related to disease severity. Overall, 8 out of 17 inflammation markers that were up-regulated in TB patients were also significantly (p<0.03 - <0.0001) elevated in mod-sev TB as well as anemic TB patients, generating an inflammation response module consisting of BAFF, LIGHT, sTNF-R1 and 2, IP-10, osteopontin, CHI3L1 and IFNα2 (**Figures 5A-F**; **Table S1**). APRIL demonstrated a very similar expression profile compared to BAFF (**Table S1**). Majority of these markers were significantly down-regulated upon successful chemotherapy (**Figures 5A-E**, right panel). Eotaxin levels were not restored during the first months of treatment (**Figure 5F**), but other markers that were down-modulated in the TB patients such as osteocalcin and MMP-2 were significantly up-regulated after 16 weeks chemotherapy (data not shown). Another 5 markers were only related to clinical disease severity and anemia including sCD30, IL-8, IL-20, IL-29/IFNδ1 and TNF-α (**Table S1**, **S2A, B**). Notably, CHI3L1 and IL-8 were the only markers significantly up-regulated in mod-sev TB, anemic TB as well as patients with extensive lung involvement (data not shown). BAFF and APRIL were also relatively higher in extensive compared to limited lung disease (data not shown).

**Figure 6:**
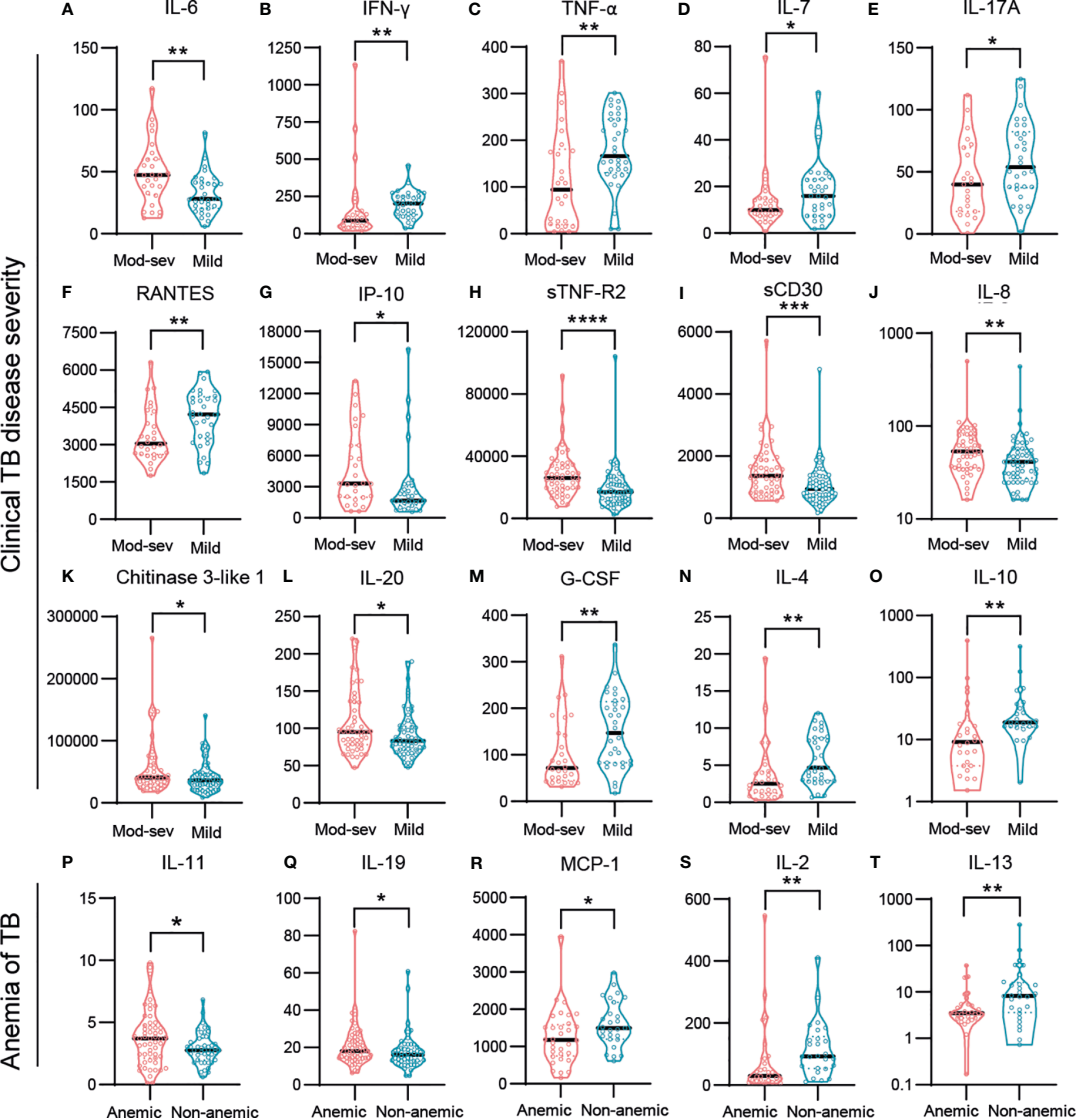
Baseline responses of selected inflammation mediators and cytokines in TB patients with different disease severity. **(A)** IL-6, **(B)** INF-γ, **(C)** TNF-α, **(D)** IL-7, **(E)** IL-17A, **(F)** RANTES, **(G)** IP-10, **(H)** sTNF-R2, **(I)** sCD30, **(J)** IL-8, **(K)** Chitinase 3-like 1, **(L)** IL-20, **(M)** G-CSF, **(N)** IL-4 **(O)** IL-10, **(P)** IL-11, **(Q)** IL-19, **(R)** MCP-1, **(S)** IL-2, **(T)** IL-13. Biomarker levels (pg/ml) are shown in patients with mod-sev TB compared to mild TB disease **(A-O)**, and in anemic TB patients compared to non-anemic TB patients **(P-T)**. Data is presented in violin plots (median and range) and was analyzed using a Mann-Whitney *U* test or an unpaired t-test. *p≤0.05, **p≤0.01, ***p≤0.001, and ****p≤0.0001.

Finally, to obtain an overview of markers related with potential protective or harmful effects, we mapped the immune markers associated with clinical TB disease severity and anemia (**Figures 6A-T**). While pro-inflammatory IL-6 (p<0.009) was elevated in the mod-sev TB group, Th1 cytokines such as IFN-γ (p<0.002) and TNF-α (p<0.009) but also IL-7 (p<0.05), IL-17A (p<0.05) and RANTES (p<0.009), were all significantly higher in mild TB disease (**Figures 6A-F**). Interestingly, both IP-10 (p<0.02), sTNF-R1 (p<0.03) and sTNF-R2 (p<0.0001) (**Figures 5C, D, G, H**), were inversely expressed as compared to IFN-γ and TNF-α (**Figures 6B, C**). Likewise, sCD30 (p<0.0005), IL-8 (p<0.005), CHI3L1 (p<0.04) and IL-20 (p<0.03) were significantly higher in mod-sev TB disease, while anti-inflammatory cytokines G-CSF (p<0.007), IL-4 (p<0.003) and IL-10 (p<0.005) were clearly higher in mild TB disease (**Figures 6I-O**). In support of the results in mod-sev TB, findings comparing anemic to non-anemic TB patients suggested that the immunomodulatory cytokines IL-11 (p<0.03) and IL-19 (p<0.04) were higher in TB patients with anemia, and IL-2 (p<0.006), MCP-1 (p<0.03) as well as IL-13 (p<0.009) were elevated in non-anemic disease (**Figures 6P-T**). Overall, these results implicate a mixed Th1/Th2 response in TB patients with mild disease, whereas inflammatory mediators and cytokines that are involved in pathological inflammation were more prevalent in mod-sev TB disease.

## 4 Discussion

### 4.1 Summary of current findings

This study intended to explore the peripheral inflammation profile in sub-groups of TB patients based on clinical disease severity, presence of anemia, or the radiological extent of lung disease. Multiplex protein analyses in plasma samples and QuantiFERON cell culture supernatants from TB patients and healthy controls demonstrated that distinct clusters of inflammation markers and cytokines separated the different TB disease sub-groups with variable efficacy. The sets of top-ranked markers changed to a variable degree depending on the inclusion of the healthy controls. Accordingly, a distinct set of markers may be effective to discriminate between TB patients and controls, while other markers may be effective to separate different disease severity groups or to follow treatment response over time. While several markers overlapped upon comparison of different TB patient sub-groups, other markers were unique with regards to their importance to differentiate the TB disease severity groups. The clinical disease and anemia sub-groups were generally more coherent compared to TB patients grouped based on the extent of lung involvement. Clearly, an immune response profile defined by up-regulated levels of BAFF, LIGHT, sTNF-R1 and 2, IP-10, osteopontin, CHI3L1 and IFNα2 and IL-8, were effective in separating TB patients with different disease clinical severity and were also rapidly down-regulated after start of anti-TB treatment. In addition, TB patients with mild disease displayed immune polarization towards mixed Th1/Th2 responses, while IL-6, IL-8, and the IL-20 subfamily of cytokines were more predominant in mod-sev TB disease and anemia of TB. Altogether, these results contribute to the understanding of the pathophysiology in TB and how to define peripheral immune response profiles to assess disease severity and to follow treatment prognosis and outcome of TB disease.

### 4.2 Discrepancy between clinical TB scores and radiological findings

A strength of this study is the sub-group comparisons that allowed us to view immunomodulation and the extent of inflammation in TB disease more carefully. For this purpose, we used a well-characterized patient cohort where clinical disease severity was determined using numerical scoring of typical TB symptoms according to a previously validated method (23–25). Many patients were malnourished and anemic and had low peripheral CD4 T cells counts and low albumin levels, consistent with progressive TB disease. However, clinical TB disease and anemia did not correlate with the extent of pulmonary involvement as determined with chest X-ray. Radiological manifestations in TB are heterogeneous, and it is not clear how clinical symptoms are associated to the extent of lung involvement at the time of diagnosis. Although, it is evident that cavitary TB is related to a poor prognosis including unfavorable treatment outcomes, treatment relapse, higher transmission rates, and development of drug resistance (31). The data from this study demonstrated that chronic inflammation in anemic TB patients was a highly associated to more severe clinical TB disease, which support the notion that anemia is a better predictor of disease severity compared to chest X-ray findings such as cavitation in TB. A previous study comparing pulmonary TB patients with and without diabetes (DM) demonstrated that a clinical score was not different comparing TB to TBDM patients, while radiological data revealed that cavitation was significantly more common in TBDM patients (32). Therefore, clinical disease severity and the extent of lung involvement may not be entirely consistent. In addition, it was found that TB and TBDM patient groups displayed similar cure rates with anti-TB therapy, despite a higher presence of cavitary TB among DM patients (32). Various methods and scoring systems to assess and quantify radiological changes in the lung have been reported that include different parameters such as cavitation, numbers and types of lesions, location, size, and coverage (33–35). PET-CT likely provides a more accurate image of the pathological involvement, but is not available at all health care facilities, especially not in developing countries.

### 4.3 Use of multiplex assays to explore immune biomarkers in TB infection and disease

To our knowledge, this is the first investigation to map a larger number of inflammatory mediators and cytokines based on clinical TB disease severity and anemia. Numerous studies have been conducted to investigate the diagnostic and prognostic potential of cytokines and chemokines in TB as compared to healthy controls and/or individuals with latent TB (36–40) and as biomarkers of TB disease severity and outcome (41, 42). A recent study compared a 14-plex cytokine assay in patients with drug-susceptible or multidrug-resistant (MDR)-TB as well as individuals with latent TB and uninfected controls and reported a mixed Th1/Th17/Th2 response in the MDR-TB patients with cytokine levels significantly higher compared to the other groups (40). This was proposed as a signature of hyperinflammation and disease severity that could discriminate different stages of Mtb infection (40). However, no significant differences in clinical symptoms were identified in the enrolled MDR-TB patients compared to patients with drug-susceptible TB (40), which suggests that clinical disease severity was not different in these groups. A similar study proposed that CXCL9 and IP-10 could be used as biomarkers to differentiate drug-susceptible TB from MDR-TB (43). The regulation of chemokines and chemokine receptor expression controls the recruitment of imperative effector cells that participate in granuloma formation and bacterial control, while other subsets of inflammatory cells promote pathological processes (7). Several cytokines and chemokines including IFNγ, TNFα, IL-17A, IL-1β (44) and MIP-1α and IP-10, have been described to be associated with bilateral lung involvement and cavitary TB (41). Furthermore, combinations of MIP-1α, IL-8, and IP-10, were proposed as novel biomarkers for predicting adverse treatment outcomes in pulmonary TB patients (41). Consistent with the results from our study, a clinical trial previously described that the majority of 69 biomarkers tested decreased with anti-TB treatment of pulmonary TB patients, except for osteocalcin, MCP-1 and MCP-4, which were significantly increased (42). While the role of osteocalcin in TB is unknown, a cohort study demonstrated an increased risk of osteoporosis in patients with active TB that is likely caused by persistent inflammation (45). MCP-1 levels in plasma have previously been reported to increase in pulmonary TB patients upon successful chemotherapy, similar to eotaxin levels that were shown to be lower in TB patients compared to the controls (36).

### 4.4 Diverse peripheral cytokine profiles representative of different TB endotypes

It is a common notion that lost immune control in TB is characterized by excess production of pro-inflammatory cytokines together with Th1 and Th17 cytokines, which result in neutrophil infiltration and bystander action of T cells that promote pathological inflammation and tissue destruction at the site of infection in the lung (46, 47). While this is true in many aspects, poor immune control in TB has also been shown to be dictated by premature expansion of anti-inflammatory macrophages or myeloid-derived suppressor cells (MDSC) as well as regulatory B and T cells (Breg and Treg) that emerge as a result of chronic inflammation (10, 48, 49). This is particularly evident in granulomatous lesions at the site of Mtb infection, where numerous recent reports from humans and non-human primates demonstrate that dysfunctional Th1/Th17 CD4+ and cytolytic CD8+ T cells responses correlate with bacterial growth and disease progression, while type 2 immunity as well as FoxP3+ Tregs and MDSCs expressing inhibitory molecules and mediators are associated with bacterial persistence (50–56). These data are all supportive of an immunosuppressive environment in the TB lesions that are driven by a hyperinflammatory response. Single-cell sequencing of Mtb granulomas in zebra fish and non-human primates, suggested that spatial organization of granulomas involving a mix of robust Th1 (IFN-γ, IL-12, IL1-β) but also counteracting Th2 (Il-4 and IL-13) responses, were associated with macrophage epithelialization and bacterial control (57). This is in line with our findings that support a mixed Th1/Th17/Th2 response in non-anemic TB patients and patients with mild TB disease. Perhaps some of the discrepancies among different reports can be explained by the fact that heterogeneity in clinical TB disease can be classified into different disease endotypes, characterized by multiple distinct molecular traits and disease mechanisms such as either immunodeficiency or pathologic excessive inflammation (58, 59).

### 4.5 TNF superfamily members regulate T cell and macrophage responses in TB

A large group of inflammatory mediators that were differentially regulated in mild compared to mod-sev TB disease belonged to the TNF superfamily. Here, LIGHT has been shown to contribute to the activation of both CD4+ and CD8+ T cells, but not to late control of Mtb infection (60), while CD30 is required for T cell activation and organization in TB granulomas. Soluble CD30 was one of the markers that did not differ between TB patients and controls but were strongly elevated in patients with mod-sev TB disease and anemia. Interestingly, children with active TB, low weight, and low blood Hb levels, had high sCD30 levels in plasma that also correlated to disease severity (61). *In vivo*, blockade of CD30-CD30L interactions on activated T cells has been shown to promote abnormal inflammation in mycobacteria-infected mice including decreased Th1 and Th17 responses (62). Thus, it is likely that excess levels of sCD30 in the circulation could bind to membrane-bound CD30L and prevent co-stimulatory interaction with CD30, which may impair essential Th1 responses and promote mycobacterial growth (63). Similarly, sTNFR-1 has been shown to be up-regulated in active TB patient’s along with IL-8 and CXCL9 (64), while virulent Mtb can inactivate TNF-α and TNF-induced apoptosis of infected cells by release of sTNF-R2 (65). The soluble forms of TNFR are induced by TNF-α itself and act as a feed-back mechanism to prevent the pathological effects of TNF-α. As an example, serum levels of sTNF-R1and 2 increased markedly after intervention of myocardial infarction and primarily sTNF-R1 appeared as an independent predictor of clinical outcomes in patients (66).

### 4.6 BAFF and APRIL signaling promote B cell responses in TB and other diseases

Our multiplex analyses demonstrated that BAFF and the related molecule APRIL, were among the top-ranked classifiers used to separate mild from mod-sev TB or non-anemic patients from anemia of TB. The BAFF/APRIL signaling pathways are known to be of crucial importance for B cell development and have a clinical relevance for development of autoimmune diseases but also infections (67, 68). Interestingly, BAFF expression is increased by IFN-α signaling (69, 70) and excess BAFF promotes inflammation in autoimmune diseases by increasing B cell numbers and antibody titers. Accordingly, neutrophils have been shown to produce BAFF that highly accelerated plasma cell generation and antigen-specific antibody production (71). It has also been reported that elevated levels of BAFF may activate class switching of B cells to enhance humoral immune responses in patients with TB pleuritis (72). A role of BAFF/APRIL on T cell function and survival has also been proposed (73) and increased levels of BAFF and APRIL mRNA were previously found in peripheral CD4+ T cells isolated from patients with active TB (74). These studies suggest that BAFF and APRIL correlate with enhanced Th1 responses and elevated survival of inflammatory CD4+ T cells (74, 75). Plasma levels of BAFF in our study correlated strongly with IL-6 (r=0.52, p<0.0001) but not with TNF-α or IFN-γ (data not shown). Moreover, while most CD19+ B cells express the BAFF-receptor, only a few percent of CD4+ and <1% of CD8+ T cells express this receptor, and majority of the BAFF-R expression was found on the surface of CD4+CD25+ Treg cells (76). These studies emphasize that the importance of elevated BAFF and APRIL levels in Mtb pathogenesis has not yet been properly addressed, but the overall relevance of B cells and humoral immunity in TB remains controversial. We have previous shown that enhanced plasmablast responses in pulmonary TB patients including antigen-specific IgG levels were associated with impaired peripheral Th1 cell responses and progressive TB disease (77). Likewise, bacterial persistence in granulomas has been shown to be related to enrichment of plasma cells, coordinated via Th2 signaling pathways (52). Possibly, B cells and antibodies may dictate Mtb-specific immune responses toward protection or pathogenesis depending on the stage of infection as well as the TB-specific endotype.

### 4.7 Immunoregulatory cytokines prevent proper activation of CD4+ T cells in a diverse spectrum of diseases including TB

Several cytokines in the multiplex assays tested exhibited anti-inflammatory and/or immunomodulatory effects. Comparisons of patients with mild TB and mod-sev disease or anemia, demonstrated that Th1 (IL-2, IFN-γ, and TNF-α) as well as Th2 (IL-4, IL-13, and IL-10) and Th17 responses were higher in mild TB disease. Instead, mod-sev TB disease presented higher levels of Osteopontin, CHI3L1, IL-11, IL-19 and IL-20. It has previously been shown that the profibrogenic molecule, Osteopontin, is elevated in TB patients (78) and that this could be considered as a potential biomarker for TB surveillance and severity assessment (19). CHI3L1 is a another profibrogenic factor that has been strongly associated with diseases including asthma, arthritis, sepsis, diabetes, liver fibrosis, and is also involved in cancer cell growth and proliferation including activation of tumor-associated macrophages, and Th2 polarization of CD4+ T cells (79). Likewise, G-CSF stimulation of PBMCs *in vitro*, alters the T cell function and promotes a Th2 type with an increase of IL-4 and decrease of IFN-α production (80). Accordingly, skewing towards a Th2 response in patients with cystic fibrosis and *P. aeruginosa* infection, correlated with elevated serum levels of G-CSF (81). In the human lung, IL-11 upregulation has been associated with a range of fibroinflammatory diseases, and fibroblast-specific IL-11 signaling drives chronic inflammation in fibrotic lung disease in mice (15). As such, a pathogenic role of IL-11 in TB infection has been proposed to involve early lung inflammation including pro-inflammatory cytokines and neutrophilic infiltration (82). The IL-20 family of cytokines are mainly expressed by lung epithelial cells that may dampen inflammatory responses during chronic inflammation. While the role of IL-20 cytokines in TB is poorly investigated, one report demonstrated that IL-19 and IL-24 are elevated in pulmonary TB patients and *in vitro* neutralization of these cytokines resulted in an enhanced CD4+ Th1/Th17 responses (14). Moreover, it has been found that IL-19, IL-20 and IL-24 promoted cutaneous infection with *S. aureus* in mice by down-regulation of IL-1β and IL-17A-dependent pathways (83). Inhibition of these IL-20 cytokines also improved bacterial clearance of *S. pneumoniae* and decreased pro-inflammatory cytokines and recruitment of neutrophils and dendritic cells in the lung (84). Perhaps these different immunomodulatory mediators contribute to inappropriate activation and poor recruitment of imperative immune cell subsets to the site of infection in the TB granuloma.

### 4.8 Soluble immune biomarkers as prognostic tools of TB disease and outcome

Even though the main aim with this study was to obtain new knowledge that could increase the understanding of the host immune response to Mtb, these results also add to the current literature related to discovery of biomarkers or immune response modules that could function as correlates of immune protection or progressive TB disease (85). TB disease severity is often assessed on the basis of chest X-ray or bacteriological results including sputum-positivity or numbers of acid-fast bacilli in sputum (86). In clinical trials, time to sputum conversion at 2 months is a common hard endpoint but is practically demanding to coordinate in an effective manner (87). Therefore, suitable surrogate markers in peripheral blood could improve and facilitate qualitative and large-scale assessment of TB disease status and treatment outcome (88). A recent meta-analysis on biomarkers in active TB, identified a total of 81 markers with the potential to be used in treatment monitoring (89). This review highlighted the barriers created by heterogeneity in study design patient cohorts and data reporting (89), which are difficult to overcome comparing many small sized studies. Even so, multi-omics studies on proteins in circulation also enables a comprehensive understanding of the interaction of the immune system and the bacteria and facilitates identification of immune pathways that contribute to disease development. Importantly, immune biomarkers may not only benefit routine clinical management but also assessment of randomized trials, especially in a time when research on host-directed therapies comprise great future potential as adjunct treatment options for diverse groups of TB patients (90, 91).

## Data availability statement

The original contributions presented in the study are included in the article/**Supplementary Material**. Further inquiries can be directed to the corresponding author.

## Ethics statement

The studies involving humans were approved by the regional ethical review boards in Addis Ababa, Ethiopia and Stockholm, Sweden (EPN, dnr 2011/1014-31/1). The studies were conducted in accordance with the local legislation and institutional requirements. The participants provided their written informed consent to participate in this study.

## Author contributions

SA: Conceptualization, Data curation, Formal analysis, Investigation, Methodology, Project administration, Software, Validation, Visualization, Writing – original draft. ML: Conceptualization, Data curation, Formal analysis, Investigation, Software, Visualization, Writing – original draft. AB: Conceptualization, Investigation, Methodology, Project administration, Resources, Writing – review & editing. GAs: Conceptualization, Investigation, Methodology, Writing – review & editing, Formal analysis. WA: Conceptualization, Investigation, Methodology, Writing – review & editing. EK: Conceptualization, Investigation, Methodology, Writing – review & editing. GAd: Conceptualization, Investigation, Methodology, Writing – review & editing, Project administration, Supervision. SB: Conceptualization, Investigation, Methodology, Project administration, Supervision, Data curation, Formal analysis, Funding acquisition, Resources, Software, Validation, Visualization, Writing – original draft.

## References

[1] Cadena AM Fortune SM Flynn JL. Heterogeneity in tuberculosis. Nat Rev Immunol (2017) 17(11):691–702. doi: 10.1038/nri.2017.69 28736436 PMC6247113

[2] Sasindran SJ Torrelles JB. Mycobacterium tuberculosis infection and inflammation: what is beneficial for the host and for the bacterium? Front Microbiol (2011) 2:2. doi: 10.3389/fmicb.2011.00002 21687401 PMC3109289

[3] Domingo-Gonzalez R Prince O Cooper A Khader SA. Cytokines and chemokines in mycobacterium tuberculosis infection. Microbiol Spectr (2016) 4(5):1–37. doi: 10.1128/microbiolspec.TBTB2-0018-2016 PMC5205539 27763255

[4] Tiwari D Martineau AR. Inflammation-mediated tissue damage in pulmonary tuberculosis and host-directed therapeutic strategies. Semin Immunol (2023) 65:101672. doi: 10.1016/j.smim.2022.101672 36469987

[5] Luies L du Preez I. The echo of pulmonary tuberculosis: mechanisms of clinical symptoms and other disease-induced systemic complications. Clin Microbiol Rev (2020) 33(4):1–19. doi: 10.1128/CMR.00036-20 PMC7331478 32611585

[6] O’Garra A Redford PS McNab FW Bloom CI Wilkinson RJ Berry MP. The immune response in tuberculosis. Annu Rev Immunol (2013) 31:475–527. doi: 10.1146/annurev-immunol-032712-095939 23516984

[7] Saunders BM Britton WJ. Life and death in the granuloma: immunopathology of tuberculosis. Immunol Cell Biol (2007) 85(2):103–11. doi: 10.1038/sj.icb.7100027 17213830

[8] van Rensburg IC Wagman C Stanley K Beltran C Ronacher K Walzl G. Successful TB treatment induces B-cells expressing FASL and IL5RA mRNA. Oncotarget (2017) 8(2):2037–43. doi: 10.18632/oncotarget.12184 PMC5356777 27682872

[9] Arranz-Trullen J Lu L Pulido D Bhakta S Boix E. Host antimicrobial peptides: the promise of new treatment strategies against tuberculosis. Front Immunol (2017) 8:1499. doi: 10.3389/fimmu.2017.01499 29163551 PMC5681943

[10] Brighenti S Joosten SA. Friends and foes of tuberculosis: modulation of protective immunity. J Intern Med (2018) 10:125–44. doi: 10.1111/joim.12778 PMC6258359 29804292

[11] Vazquez MI Catalan-Dibene J Zlotnik A. B cells responses and cytokine production are regulated by their immune microenvironment. Cytokine (2015) 74(2):318–26. doi: 10.1016/j.cyto.2015.02.007 PMC4475485 25742773

[12] Lu LL Chung AW Rosebrock TR Ghebremichael M Yu WH Grace PS. A functional role for antibodies in tuberculosis. Cell (2016) 167(2):433–43 e14. doi: 10.1016/j.cell.2016.08.072 27667685 PMC5526202

[13] Rutz S Wang X Ouyang W. The IL-20 subfamily of cytokines–from host defence to tissue homeostasis. Nat Rev Immunol (2014) 14(12):783–95. doi: 10.1038/nri3766 25421700

[14] Kumar NP Moideen K Banurekha VV Nair D Babu S. Modulation of Th1/Tc1 and Th17/Tc17 responses in pulmonary tuberculosis by IL-20 subfamily of cytokines. Cytokine (2018) 108:190–6. doi: 10.1016/j.cyto.2018.04.005 PMC5962435 29684756

[15] Ng B Cook SA Schafer S. Interleukin-11 signaling underlies fibrosis, parenchymal dysfunction, and chronic inflammation of the airway. Exp Mol Med (2020) 52(12):1871–8. doi: 10.1038/s12276-020-00531-5 PMC7705429 33262481

[16] Boxx GM Cheng G. The roles of type I interferon in bacterial infection. Cell Host Microbe (2016) 19(6):760–9. doi: 10.1016/j.chom.2016.05.016 PMC5847370 27281568

[17] Odendall C Voak AA Kagan JC. Type III IFNs are commonly induced by bacteria-sensing TLRs and reinforce epithelial barriers during infection. J Immunol (2017) 199(9):3270–9. doi: 10.4049/jimmunol.1700250 PMC5679450 28954888

[18] Rohlwink UK Walker NF Ordonez AA Li YJ Tucker EW Elkington PT. Matrix metalloproteinases in pulmonary and central nervous system tuberculosis-A review. Int J Mol Sci (2019) 20(6):1–35. doi: 10.3390/ijms20061350 PMC6471445 30889803

[19] Wang D Tong X Wang L Zhang S Huang J Zhang L. The association between osteopontin and tuberculosis: A systematic review and meta-analysis. PloS One (2020) 15(12):e0242702. doi: 10.1371/journal.pone.0242702 33264357 PMC7710079

[20] Ashenafi S Bekele A Aseffa G Amogne W Kassa E Aderaye G. Anemia is a strong predictor of wasting, disease severity, and progression, in clinical tuberculosis (TB). Nutrients (2022) 14(16):1–14. doi: 10.3390/nu14163318 PMC9413151 36014824

[21] Ganz T. Anemia of inflammation. N Engl J Med (2019) 381(12):1148–57. doi: 10.1056/NEJMra1804281 31532961

[22] Dasaradhan T Koneti J Kalluru R Gadde S Cherukuri SP Chikatimalla R. Tuberculosis-associated anemia: A narrative review. Cureus (2022) 14(8):e27746. doi: 10.7759/cureus.27746 36106202 PMC9447415

[23] Bekele A Gebreselassie N Ashenafi S Kassa E Aseffa G Amogne W. Daily adjunctive therapy with vitamin D(3) and phenylbutyrate supports clinical recovery from pulmonary tuberculosis: a randomized controlled trial in Ethiopia. J Intern Med (2018) 284(3):292–306. doi: 10.1111/joim.12767 29696707 PMC6202271

[24] Wejse C Gustafson P Nielsen J Gomes VF Aaby P Andersen PL. TBscore: Signs and symptoms from tuberculosis patients in a low-resource setting have predictive value and may be used to assess clinical course. Scand J Infect Dis (2008) 40(2):111–20. doi: 10.1080/00365540701558698 17852907

[25] Wejse C Gomes VF Rabna P Gustafson P Aaby P Lisse IM. Vitamin D as supplementary treatment for tuberculosis: a double-blind, randomized, placebo-controlled trial. Am J Respir Crit Care Med (2009) 179(9):843–50. doi: 10.1164/rccm.200804-567OC 19179490

[26] Nicoll D Mark LC McPhee SJ. Guide to diagnostic tests (2017). Columbus, OH, USA: McGraw Hill. Available at: https://accessmedicine.mhmedical.com/content.aspx?bookid=2032&sectionid=151444058 (Accessed 15 October 2021).

[27] Aderaye G Bruchfeld J Assefa G Feleke D Kallenius G Baat M. The relationship between disease pattern and disease burden by chest radiography, M. tuberculosis Load, and HIV status in patients with pulmonary tuberculosis in Addis Ababa. Infection (2004) 32(6):333–8. doi: 10.1007/s15010-004-3089-x 15597222

[28] Patil I. Visualizations with statistical details: The ‘ggstatsplot’ approach. J Open Source Software (2021) 6(61):3167. doi: 10.21105/joss.03167

[29] Jones BE Oo MM Taikwel EK Qian D Kumar A Maslow ER. CD4 cell counts in human immunodeficiency virus-negative patients with tuberculosis. Clin Infect Dis (1997) 24(5):988–91. doi: 10.1093/clinids/24.5.988 9142808

[30] Murthy SE Chatterjee F Crook A Dawson R Mendel C Murphy ME. Pretreatment chest x-ray severity and its relation to bacterial burden in smear positive pulmonary tuberculosis. BMC Med (2018) 16(1):73. doi: 10.1186/s12916-018-1053-3 29779492 PMC5961483

[31] Urbanowski ME Ordonez AA Ruiz-Bedoya CA Jain SK Bishai WR. Cavitary tuberculosis: the gateway of disease transmission. Lancet Infect Dis (2020) 20(6):e117–e28. doi: 10.1016/S1473-3099(20)30148-1 PMC7357333 32482293

[32] Gil-Santana L Almeida-Junior JL Oliveira CA Hickson LS Daltro C Castro S. Diabetes is associated with worse clinical presentation in tuberculosis patients from Brazil: A retrospective cohort study. PloS One (2016) 11(1):e0146876. doi: 10.1371/journal.pone.0146876 26752596 PMC4709051

[33] Kriel M Lotz JW Kidd M Walzl G. Evaluation of a radiological severity score to predict treatment outcome in adults with pulmonary tuberculosis. Int J Tuberc Lung Dis (2015) 19(11):1354–60. doi: 10.5588/ijtld.15.0098 26467588

[34] Chakraborthy A Shivananjaiah AJ Ramaswamy S Chikkavenkatappa N. Chest X ray score (Timika score): an useful adjunct to predict treatment outcome in tuberculosis. Adv Respir Med (2018) 86(5):205–10. doi: 10.5603/ARM.2018.0032 30378646

[35] Ralph AP Ardian M Wiguna A Maguire GP Becker NG Drogumuller G. A simple, valid, numerical score for grading chest x-ray severity in adult smear-positive pulmonary tuberculosis. Thorax (2010) 65(10):863–9. doi: 10.1136/thx.2010.136242 20861290

[36] Djoba Siawaya JF Beyers N van Helden P Walzl G. Differential cytokine secretion and early treatment response in patients with pulmonary tuberculosis. Clin Exp Immunol (2009) 156(1):69–77. doi: 10.1111/j.1365-2249.2009.03875.x 19196252 PMC2673743

[37] Mihret A Bekele Y Bobosha K Kidd M Aseffa A Howe R. Plasma cytokines and chemokines differentiate between active disease and non-active tuberculosis infection. J Infect (2013) 66(4):357–65. doi: 10.1016/j.jinf.2012.11.005 23178506

[38] Zhao Y Yang X Zhang X Yu Q Zhao P Wang J. IP-10 and RANTES as biomarkers for pulmonary tuberculosis diagnosis and monitoring. Tuberculosis (Edinb) (2018) 111:45–53. doi: 10.1016/j.tube.2018.05.004 30029914

[39] Moideen K Kumar NP Bethunaickan R Banurekha VV Nair D Babu S. Heightened systemic levels of anti-inflammatory cytokines in pulmonary tuberculosis and alterations following anti-tuberculosis treatment. Cytokine (2020) 127:154929. doi: 10.1016/j.cyto.2019.154929 31811994

[40] Sampath P Rajamanickam A Thiruvengadam K Natarajan AP Hissar S Dhanapal M. Cytokine upsurge among drug-resistant tuberculosis endorse the signatures of hyper inflammation and disease severity. Sci Rep (2023) 13(1):785. doi: 10.1038/s41598-023-27895-8 36646786 PMC9842614

[41] Kumar NP Moideen K Nancy A Viswanathan V Thiruvengadam K Nair D. Plasma chemokines are baseline predictors of unfavorable treatment outcomes in pulmonary tuberculosis. Clin Infect Dis (2021) 73(9):e3419–e27. doi: 10.1093/cid/ciaa1104 PMC8563183 32766812

[42] Sigal GB Segal MR Mathew A Jarlsberg L Wang M Barbero S. Biomarkers of tuberculosis severity and treatment effect: A directed screen of 70 host markers in a randomized clinical trial. EBioMedicine (2017) 25:112–21. doi: 10.1016/j.ebiom.2017.10.018 PMC5704068 29100778

[43] Sampath P Rajamanickam A Thiruvengadam K Natarajan AP Hissar S Dhanapal M. Plasma chemokines CXCL10 and CXCL9 as potential diagnostic markers of drug-sensitive and drug-resistant tuberculosis. Sci Rep (2023) 13(1):7404. doi: 10.1038/s41598-023-34530-z 37149713 PMC10163852

[44] Kumar NP Moideen K Banurekha VV Nair D Babu S. Plasma proinflammatory cytokines are markers of disease severity and bacterial burden in pulmonary tuberculosis. Open Forum Infect Dis (2019) 6(7):ofz257. doi: 10.1093/ofid/ofz257 31281858 PMC6602384

[45] Feng JY Chen YY Yen YF Pan SW Su WJ. Active tuberculosis increases the risk of incident osteoporosis- A nation-wide population based cohort study. Eur Respir J (2016) 48:OA4823. doi: 10.1183/13993003.congress-2016.OA4823

[46] Kaufmann SH Dorhoi A. Inflammation in tuberculosis: interactions, imbalances and interventions. Curr Opin Immunol (2013) 25(4):441–9. doi: 10.1016/j.coi.2013.05.005 23725875

[47] Muefong CN Sutherland JS. Neutrophils in tuberculosis-associated inflammation and lung pathology. Front Immunol (2020) 11:962. doi: 10.3389/fimmu.2020.00962 32536917 PMC7266980

[48] Shaw JA Malherbe ST Walzl G du Plessis N. Suppressive myeloid cells in SARS-CoV-2 and Mycobacterium tuberculosis co-infection. Front Immunol (2023) 14:1222911. doi: 10.3389/fimmu.2023.1222911 37545508 PMC10399583

[49] Ashenafi S Brighenti S. Reinventing the human tuberculosis (TB) granuloma: Learning from the cancer field. Front Immunol (2022) 13:1059725. doi: 10.3389/fimmu.2022.1059725 36591229 PMC9797505

[50] McCaffrey EF Donato M Keren L Chen Z Delmastro A Fitzpatrick MB. The immunoregulatory landscape of human tuberculosis granulomas. Nat Immunol (2022) 23(2):318–29. doi: 10.1038/s41590-021-01121-x PMC8810384 35058616

[51] Singh B Singh DK Ganatra SR Escobedo RA Khader S Schlesinger LS. Myeloid-derived suppressor cells mediate T cell dysfunction in nonhuman primate TB granulomas. mBio (2021) 12(6):e0318921. doi: 10.1128/mbio.03189-21 34903057 PMC8669465

[52] Gideon HP Hughes TK Tzouanas CN Wadsworth MH 2nd Tu AA Gierahn TM. Multimodal profiling of lung granulomas in macaques reveals cellular correlates of tuberculosis control. Immunity (2022) 55(5):827–46 e10. doi: 10.1016/j.immuni.2022.04.004 35483355 PMC9122264

[53] Esaulova E Das S Singh DK Choreno-Parra JA Swain A Arthur L. The immune landscape in tuberculosis reveals populations linked to disease and latency. Cell Host Microbe (2021) 29(2):165–78 e8. doi: 10.1016/j.chom.2020.11.013 33340449 PMC7878437

[54] Rahman S Gudetta B Fink J Granath A Ashenafi S Aseffa A. Compartmentalization of immune responses in human tuberculosis: few CD8+ effector T cells but elevated levels of FoxP3+ regulatory t cells in the granulomatous lesions. Am J Pathol (2009) 174(6):2211–24. doi: 10.2353/ajpath.2009.080941 PMC2684186 19435796

[55] Carow B Hauling T Qian X Kramnik I Nilsson M Rottenberg ME. Spatial and temporal localization of immune transcripts defines hallmarks and diversity in the tuberculosis granuloma. Nat Commun (2019) 10(1):1823. doi: 10.1038/s41467-019-09816-4 31015452 PMC6479067

[56] Ashenafi S Muvva JR Mily A Snall J Zewdie M Chanyalew M. Immunosuppressive features of the microenvironment in lymph nodes granulomas from tuberculosis and HIV-co-infected patients. Am J Pathol (2022) 192(4):653–70. doi: 10.1016/j.ajpath.2021.12.013 PMC9302207 35092727

[57] Cronan MR Hughes EJ Brewer WJ Viswanathan G Hunt EG Singh B. A non-canonical type 2 immune response coordinates tuberculous granuloma formation and epithelialization. Cell (2021) 184(7):1757–74 e14. doi: 10.1016/j.cell.2021.02.046 33761328 PMC8055144

[58] DiNardo AR Gandhi T Heyckendorf J Grimm SL Rajapakshe K Nishiguchi T. Gene expression signatures identify biologically and clinically distinct tuberculosis endotypes. Eur Respir J (2022) 60(3):1–15. doi: 10.1183/13993003.02263-2021 PMC9474892 35169026

[59] DiNardo AR Nishiguchi T Grimm SL Schlesinger LS Graviss EA Cirillo JD. Tuberculosis endotypes to guide stratified host-directed therapy. Med (2021) 2(3):217–32. doi: 10.1016/j.medj.2020.11.003 PMC8530349 34693385

[60] Musicki K Briscoe H Britton WJ Saunders BM. LIGHT contributes to early but not late control of Mycobacterium tuberculosis infection. Int Immunol (2010) 22(5):353–8. doi: 10.1093/intimm/dxq013 20185431

[61] Hanekom WA Hussey GD Hughes EJ Potgieter S Yogev R Check IJ. Plasma-soluble CD30 in childhood tuberculosis: effects of disease severity, nutritional status, and vitamin A therapy. Clin Diagn Lab Immunol (1999) 6(2):204–8. doi: 10.1128/CDLI.6.2.204-208.1999 PMC95688 10066655

[62] Marin ND Garcia LF. The role of CD30 and CD153 (CD30L) in the anti-mycobacterial immune response. Tuberculosis (Edinb) (2017) 102:8–15. doi: 10.1016/j.tube.2016.10.006 28061955

[63] Tang C Yamada H Shibata K Muta H Wajjwalku W Podack ER. A novel role of CD30L/CD30 signaling by T-T cell interaction in Th1 response against mycobacterial infection. J Immunol (2008) 181(9):6316–27. doi: 10.4049/jimmunol.181.9.6316 18941223

[64] Alessandri AL Souza AL Oliveira SC Macedo GC Teixeira MM Teixeira AL. Concentrations of CXCL8, CXCL9 and sTNFR1 in plasma of patients with pulmonary tuberculosis undergoing treatment. Inflammation Res (2006) 55(12):528–33. doi: 10.1007/s00011-006-5136-9 17039284

[65] Balcewicz-Sablinska MK Keane J Kornfeld H Remold HG. Pathogenic Mycobacterium tuberculosis evades apoptosis of host macrophages by release of TNF-R2, resulting in inactivation of TNF-alpha. J Immunol (1998) 161(5):2636–41. doi: 10.4049/jimmunol.161.5.2636 9725266

[66] Paccalet A Crola Da Silva C Mechtouff L Amaz C Varillon Y de Bourguignon C. Serum soluble tumor necrosis factor receptors 1 and 2 are early prognosis markers after ST-segment elevation myocardial infarction. Front Pharmacol (2021) 12:656928. doi: 10.3389/fphar.2021.656928 34539391 PMC8440863

[67] Vincent FB Saulep-Easton D Figgett WA Fairfax KA Mackay F. The BAFF/APRIL system: emerging functions beyond B cell biology and autoimmunity. Cytokine Growth Factor Rev (2013) 24(3):203–15. doi: 10.1016/j.cytogfr.2013.04.003 PMC7108297 23684423

[68] Sakai J Akkoyunlu M. The role of BAFF system molecules in host response to pathogens. Clin Microbiol Rev (2017) 30(4):991–1014. doi: 10.1128/CMR.00046-17 28855265 PMC5608883

[69] Panchanathan R Choubey D. Murine BAFF expression is up-regulated by estrogen and interferons: implications for sex bias in the development of autoimmunity. Mol Immunol (2013) 53(1-2):15–23. doi: 10.1016/j.molimm.2012.06.013 22784990 PMC3439561

[70] Mourik BC Lubberts E de Steenwinkel JEM Ottenhoff THM Leenen PJM. Interactions between type 1 interferons and the th17 response in tuberculosis: lessons learned from autoimmune diseases. Front Immunol (2017) 8:294. doi: 10.3389/fimmu.2017.00294 28424682 PMC5380685

[71] Parsa R Lund H Georgoudaki AM Zhang XM Ortlieb Guerreiro-Cacais A Grommisch D. BAFF-secreting neutrophils drive plasma cell responses during emergency granulopoiesis. J Exp Med (2016) 213(8):1537–53. doi: 10.1084/jem.20150577 PMC4986521 27432941

[72] Wang X Liang KD Zhang JA Liu GB Chen Z Chen C. Increased B cell activating factor is associated with B cell class switching in patients with tuberculous pleural effusion. Mol Med Rep (2018) 18(2):1704–9. doi: 10.3892/mmr.2018.9073 29845274

[73] Mackay F Leung H. The role of the BAFF/APRIL system on T cell function. Semin Immunol (2006) 18(5):284–9. doi: 10.1016/j.smim.2006.04.005 16931039

[74] Liu K Zhang Y Hu S Yu Y Yang Q Jin D. Increased levels of BAFF and APRIL related to human active pulmonary tuberculosis. PloS One (2012) 7(6):e38429. doi: 10.1371/journal.pone.0038429 22719887 PMC3373577

[75] Sutherland AP Ng LG Fletcher CA Shum B Newton RA Grey ST. BAFF augments certain Th1-associated inflammatory responses. J Immunol (2005) 174(9):5537–44. doi: 10.4049/jimmunol.174.9.5537 15843552

[76] Ye Q Wang L Wells AD Tao R Han R Davidson A. BAFF binding to T cell-expressed BAFF-R costimulates T cell proliferation and alloresponses. Eur J Immunol (2004) 34(10):2750–9. doi: 10.1002/eji.200425198 15368291

[77] Ashenafi S Aderaye G Zewdie M Raqib R Bekele A Magalhaes I. BCG-specific IgG-secreting peripheral plasmablasts as a potential biomarker of active tuberculosis in HIV negative and HIV positive patients. Thorax (2013) 68(3):269–76. doi: 10.1136/thoraxjnl-2012-201817 PMC3585487 22923457

[78] Nau GJ Guilfoile P Chupp GL Berman JS Kim SJ Kornfeld H. A chemoattractant cytokine associated with granulomas in tuberculosis and silicosis. Proc Natl Acad Sci U S A. (1997) 94(12):6414–9. doi: 10.1073/pnas.94.12.6414 PMC21064 9177232

[79] Zhao T Su Z Li Y Zhang X You Q. Chitinase-3 like-protein-1 function and its role in diseases. Signal Transduct Target Ther (2020) 5(1):201. doi: 10.1038/s41392-020-00303-7 32929074 PMC7490424

[80] Sloand EM Kim S Maciejewski JP Van Rhee F Chaudhuri A Barrett J. Pharmacologic doses of granulocyte colony-stimulating factor affect cytokine production by lymphocytes in *vitro* and in vivo. Blood (2000) 95(7):2269–74. doi: 10.1182/blood.V95.7.2269 10733495

[81] Moser C Jensen PO Pressler T Frederiksen B Lanng S Kharazmi A. Serum concentrations of GM-CSF and G-CSF correlate with the Th1/Th2 cytokine response in cystic fibrosis patients with chronic Pseudomonas aeruginosa lung infection. APMIS (2005) 113(6):400–9. doi: 10.1111/j.1600-0463.2005.apm_142.x 15996157

[82] Kapina MA Shepelkova GS Avdeenko VG Guseva AN Kondratieva TK Evstifeev VV. Interleukin-11 drives early lung inflammation during Mycobacterium tuberculosis infection in genetically susceptible mice. PloS One (2011) 6(7):e21878. doi: 10.1371/journal.pone.0021878 21789190 PMC3137601

[83] Myles IA Fontecilla NM Valdez PA Vithayathil PJ Naik S Belkaid Y. Signaling via the IL-20 receptor inhibits cutaneous production of IL-1beta and IL-17A to promote infection with methicillin-resistant Staphylococcus aureus. Nat Immunol (2013) 14(8):804–11. doi: 10.1038/ni.2637 PMC3721434 23793061

[84] Madouri F Barada O Kervoaze G Trottein F Pichavant M Gosset P. Production of Interleukin-20 cytokines limits bacterial clearance and lung inflammation during infection by Streptococcus pneumoniae. EBioMedicine (2018) 37:417–27. doi: 10.1016/j.ebiom.2018.10.031 PMC6284423 30361066

[85] Walzl G Ronacher K Hanekom W Scriba TJ Zumla A. Immunological biomarkers of tuberculosis. Nat Rev Immunol (2011) 11(5):343–54. doi: 10.1038/nri2960 21475309

[86] Babu S. Biomarkers for treatment monitoring in tuberculosis: A new hope. EBioMedicine (2017) 26:13–4. doi: 10.1016/j.ebiom.2017.11.002 PMC5832559 29129701

[87] Rockwood N du Bruyn E Morris T Wilkinson RJ. Assessment of treatment response in tuberculosis. Expert Rev Respir Med (2016) 10(6):643–54. doi: 10.1586/17476348.2016.1166960 PMC4949330 27030924

[88] Nahid P Saukkonen J Mac Kenzie WR Johnson JL Phillips PP Andersen J. CDC/NIH Workshop. Tuberculosis biomarker and surrogate endpoint research roadmap. Am J Respir Crit Care Med (2011) 184(8):972–9. doi: 10.1164/rccm.201105-0827WS PMC3208659 21737585

[89] Zimmer AJ Lainati F Aguilera Vasquez N Chedid C McGrath S Benedetti A. Biomarkers that correlate with active pulmonary tuberculosis treatment response: a systematic review and meta-analysis. J Clin Microbiol (2022) 60(2):e0185921. doi: 10.1128/jcm.01859-21 34911364 PMC8849205

[90] Wallis RS Maeurer M Mwaba P Chakaya J Rustomjee R Migliori GB. Tuberculosis–advances in development of new drugs, treatment regimens, host-directed therapies, and biomarkers. Lancet Infect Dis (2016) 16(4):e34–46. doi: 10.1016/S1473-3099(16)00070-0 27036358

[91] Guler R Ozturk M Sabeel S Motaung B Parihar SP Thienemann F. Targeting molecular inflammatory pathways in granuloma as host-directed therapies for tuberculosis. Front Immunol (2021) 12:733853. doi: 10.3389/fimmu.2021.733853 34745105 PMC8563828

