# Supplementary material for: Inflammatory immune profiles associated with disease severity in pulmonary tuberculosis patients with moderate to severe clinical TB or anemia
Source: Front Immunol. 2023 Dec 12;14:1296501. doi: 10.3389/fimmu.2023.1296501 (PMC10756900; doi:10.3389/fimmu.2023.1296501)
Supplement: Supplementary file 1 [file DataSheet_1.docx]

Supplementary Material

**Inflammatory immune profiles associated with disease severity in pulmonary tuberculosis (TB) patients with moderate to severe clinical TB or anemia**

**Senait Ashenafi^1,4†^, Marco Giulio Loreti^4†^, Amsalu Bekele^2^, Getachew Aseffa^3^, Wondwossen Amogne^2^, Endale Kassa^2^, Getachew Aderaye^2^, and Susanna Brighenti^4^**

^1^Department of Pathology, School of Medicine, College of Health Sciences, Tikur Anbessa Specialized Hospital and Addis Ababa University, Addis Ababa, Ethiopia

^2^Department of Internal Medicine, School of Medicine, College of Health Sciences, Tikur Anbessa Specialized Hospital and Addis Ababa University, Addis Ababa, Ethiopia

^3^Department of Radiology, School of Medicine, College of Health Sciences, Tikur Anbessa Specialized Hospital and Addis Ababa University, Addis Ababa, Ethiopia

^4^Department of Medicine Huddinge, Center for Infectious Medicine (CIM), ANA Futura, Karolinska Institutet, Stockholm, Sweden

† Equal contributions.

*** Correspondence:** Susanna Brighenti: [susanna.brighenti@ki.se](mailto:susanna.brighenti@ki.se)

# Supplementary Tables 1, 2A-B (see pages 2-4), Table 3 (see separate excel file), and Figure 1 (see page 5)

| **SUPPLEMENTARY TABLE 1 Human inflammation 37-plex panel** | | | | | | |
| --- | --- | --- | --- | --- | --- | --- |
| Markers^a^ | TB | Controls | Mod-sev TB | Mild TB | Anemic TB | Non-anemic TB |
| April/TNFSF13 | **118303.62**** | 80037.85 | 124742.64 | 112444.66 | **136410.8**** | 107410.4 |
| BAFF/TNFSF13B | **22495.53****** | 8808.095 | **28036.47****** | 16590.72 | **28036.47**** | 16590.72 |
| sCD30/TNFSF8 | 1118.02 | 821.76 | **1360.27***** | 945.695 | **1329.54***** | 966.535 |
| sCD163 | **4083.46***** | 2405.025 | 3519.98 | 4149.705 | 4077.09 | 4113.385 |
| Chitinase 3-like1 | **37714.55***** | 17514.45 | **40740.21*** | 36007.86 | **41741.855*** | 33892.8 |
| gp130/sIL-6R-β | 109271.57 | 103446.9 | 113375.02 | 109124.24 | **111932.91*** | 109253.265 |
| IFN-alpha2 | **54.64**** | 43.3 | **57.76*** | 51.61 | **59.51**** | 49.855 |
| IFN-beta | 134.43 | 121.19 | 142.94 | 132.06 | **139.31*** | 126.565 |
| IFN-γ | **43.45*** | 33.215 | 44.24 | 41.945 | 44.55 | 41.35 |
| IL-2 | 5.63 | 5.57 | 6.31 | 5.5 | 6.31 | 5.5 |
| sIL-6Rα | 16901.8 | 17211.06 | 16210.39 | 17563.35 | 17385.33 | 16393.55 |
| IL-8 | 46.68 | 32.54 | **53.36**** | 41.18 | **50.54*** | 38.465 |
| IL-10 | 9.12 | 8.34 | 8.82 | 9.27 | 8.61 | 9.42 |
| IL-11 | **3.04*** | 2.17 | 2.95 | 3.085 | **3.7*** | 2.76 |
| IL-12(p40) | 70.405 | 67.425 | 73.89 | 69.73 | 73.01 | 70.405 |
| IL-12(p70) | 0.64 | 0.435 | 0.67 | 0.6 | 0.63 | 0.67 |
| IL-19 | 17.1 | 19.4 | 17.41 | 16.95 | **18.06*** | 6.165 |
| IL-20 | 85.94 | 78.56 | **95.01*** | 83.01 | **90.06*** | 83.01 |
| IL-22 | 29.51 | 25.63 | **30.47*** | 28.51 | 31.71 | 28.49 |
| IL-26 | 29.19 | 29.465 | 29.72 | 29.185 | 31.96 | 28.225 |
| IL-27 (p28) | too low | too low | too low | too low | too low | too low |
| IL-28A/IFN-delta2 | **47.23*** | 40.435 | 47.66 | 45.92 | 49.58 | 45.92 |
| IL-29/IFN-delta1 | 34 | 33.58 | **38.505*** | 32.56 | **38.505*** | 32.56 |
| IL-32 | 75.535 | 53.84 | 78.87 | 72.34 | 75.23 | 78.52 |
| IL-34 | too low | too low | too low | too low | too low | too low |
| IL-35 | 243.09 | 208.695 | 255.1 | 241.24 | 260.25 | 234.555 |
| LIGHT/TNFSF14 | **125.1****** | 38.465 | **159.45*** | 112.35 | **152.29*** | 117.225 |
| MMP-1 | **3871.7****** | 1945.15 | 3891.33 | 3855.525 | **4095.35*** | 3270.145 |
| MMP-2 | 44380.11 | **92047.05****** | 42798.16 | 44894.08 | 42131.13 | 47482.185 |
| MMP-3 | 8217.59 | **10204.61*** | 8604.65 | 7865.08 | **10214.15**** | 6557.075 |
| Osteocalcin | 6538.48 | **9051.9**** | 6492.3 | 6842.58 | 6538.48 | 6567.84 |
| Osteopontin (OPN) | **139444.86***** | 97354.945 | **156687.15**** | 120207.615 | **149780.97**** | 118197.345 |
| Pentraxin-3 | **1551.02*** | 1190.3 | **1715.69*** | 1490.4 | 1715.69 | 1522.935 |
| sTNF-R1 | **5200.14****** | 3013.74 | **5670.44***** | 4341.6 | **5701.11****** | 4064.525 |
| sTNF-R2 | **21001.87****** | 8591.78 | **26161.31****** | 17003.115 | **24519.23**** | 17059.71 |
| TSLP | 54.35 | 54.465 | 56.99 | 54.27 | **59.96**** | 52.055 |
| TWEAK/TNFSF12 | 576.15 | 622.975 | 582.31 | 574.565 | 582.31 | 572.83 |

^a^ Data is presented as median. Statistical difference between the different sub-groups indicated by the columns was determined using a Mann-Whitney *U* test or an unpaired t-test. Statistically significant values are highlighted in bold.

Marker abbreviations: TNFSF, tumor necrosis factor super family; April, A proliferation inducing ligand; BAFF, B-cell activating factor; CD, cluster of differentiation; gp, glycoprotein; IL, interleukin; IFN, interferon; MMP, matrix metalloproteinase; sTNF-R, soluble TNF receptor; TSLP, thymic stromal lymphopoietin; TWEAK, TNF related weak inducer of apoptosis

| **SUPPLEMENTARY TABLE 2A Human cytokine 27-plex panel (plasma)** | | | | | | |
| --- | --- | --- | --- | --- | --- | --- |
| Markers | TB | Controls | Mod-sev TB | Mild TB | Anemic TB | Non-anemic TB |
| IL-1β | 6.995 | 6.31 | 5.135 | **10.115*** | 6.72 | 7.225 |
| IL-1RA | 224.305 | 199.57 | 194.935 | 286.185 | 208.385 | 261.875 |
| IL-2 | too low | too low | too low | too low | too low | too low |
| IL-4 | 3.62 | 4.14 | 2.525 | **4.67**** | 3.075 | 4.035 |
| IL-5 | too low | too low | too low | too low | too low | too low |
| IL-6 | **39.34****** | 14 | **47.385*** | 28.485 | 40.43 | 35.03 |
| IL-7 | 6.41 | 8.06 | 6.41 | 10.54 | 6.41 | 10.54 |
| IL-8 | 38.895 | 25.83 | 30.535 | 41.335 | 25.08 | 41.335 |
| IL-9 | 47.03 | 48.47 | 52.71 | 44.61 | 47.43 | 46.83 |
| IL-10 | 16.34 | 16.57 | 9.22 | **18.9**** | 12.99 | 17.3 |
| IL-12 (p70) | too low | too low | too low | too low | too low | too low |
| IL-13 | too low | too low | too low | too low | too low | too low |
| IL-15 | too low | too low | too low | too low | too low | too low |
| GM-CSF | too low | too low | too low | too low | too low | too low |
| IL-17A | 46.42 | 58.24 | 39.93 | 53.905 | 39.34 | 59.43 |
| Eotaxin | 82.9 | **163.21****** | 57.07 | **104.18**** | 71.995 | 85.82 |
| FGF basic | too low | too low | too low | too low | too low | too low |
| G-CSF | 101.57 | 113.88 | 71.65 | **147.02**** | 84.92 | 121.055 |
| IFN-γ | 146.42 | 159.63 | 87.81 | **203.685**** | 129.72 | 173.04 |
| IP-10 | **2312.905****** | 473.47 | **3301.66*** | 1669.435 | **3156.825*** | 1669.435 |
| MCP-1 | too low | too low | too low | too low | too low | too low |
| MIP-1α | 5.52 | 5.16 | 4.995 | 6.255 | 4.825 | **7.11*** |
| PDGF-bb | 502.205 | 341.34 | 478.83 | 527.53 | 494.565 | 527.53 |
| MIP-1β | 89.08 | 89.89 | 96.295 | 82.44 | 89.795 | 86.835 |
| RANTES | 3767.06 | 3721.63 | 3048.755 | **4215.565**** | 3361.77 | 4169.17 |
| TNF-α | 152.75 | 147.8 | 108.1 | **166.385*** | 125.48 | **166.295*** |
| VEGF | **62.84***** | 35.19 | 67.13 | 61.705 | 71.82 | 57.8 |

^a^ Data is presented as median. Statistical difference between the different sub-groups indicated by the columns was determined using a Mann-Whitney *U* test or an unpaired t-test. Statistically significant values are highlighted in bold.

Marker abbreviations: IL, interleukin; GM-CSF, granulocyte macrophage-colony stimulating factor; FGF, fibroblast growth factor; G-CSF, granulocyte-colony stimulating factor; IFN-γ, interferon gamma; IP-10, interferon gamma inducible protein-10; MCP-1, monocyte chemoattractant protein-1; MIP-1, macrophage inflammatory protein; PDGF, platelet derived growth factor; RANTES, regulated on activation normal T cell expressed and secreted; TNF, tumor necrosis factor; VEGF, vascular endothelial growth factor.

| **SUPPLEMENTARY TABLE 2B** **Human cytokine 27-plex panel (QuantiFERON supernatant)** | | | | | | |
| --- | --- | --- | --- | --- | --- | --- |
| Markers | TB | Controls | Mod-sev TB | Mild TB | Anemic TB | Non-anemic TB |
| IL-1β | 560.01 | 387.83 | **756.315*** | 461.73 | 615.81 | 511.9 |
| IL-1-RA | 2944.4 | 2248.74 | 2178.435 | 3244.765 | 1880.52 | **3470.2**** |
| IL-2 | 61.45 | 51.94 | 48.29 | 88.795 | 29.31 | **92.335**** |
| IL-4 | 13.865 | 13.91 | 14.525 | 13.335 | 12.625 | 14.545 |
| IL-5 | 3.51 | 4.03 | 3.495 | 3.82 | 3.51 | 4.265 |
| IL-6 | 3879.46 | 2996.55 | 5475.545 | 3538.57 | 3425.545 | 4889.88 |
| IL-7 | 13.3 | 16.89 | 10.83 | 16.975 | 12.3 | 17.865 |
| IL-8 | 25795.62 | 25714.12 | 25828.08 | 25763.15 | 21638.43 | 30226.46 |
| IL-9 | 215.425 | 217.24 | 204.62 | 225.24 | 204.62 | 219.41 |
| IL-10 | 52.665 | 42.15 | 59.095 | 42.42 | 49.84 | 54.66 |
| IL-12 (p70) | 40.435 | 50.71 | 40.98 | 40.435 | 40.435 | 41.875 |
| IL-13 | 4.57 | 5.45 | 3.68 | 5.235 | 3.5 | **8.26**** |
| IL-15 | 167.885 | 200.71 | 141.46 | 184.8 | 141.46 | 185.8 |
| GM-CSF | too low | too low | too low | too low | too low | too low |
| IL-17A | 434.175 | 493.07 | 397.86 | 513.28 | 377.595 | **508.59*** |
| Eotaxin | 172.9 | **239.05***** | 177.49 | 165.235 | 176.515 | 165.235 |
| FGF basic | 99.075 | 93.235 | 86.85 | 109.52 | 82.16 | 109.235 |
| G-CSF | 327.26 | 396.87 | 363.785 | 304.345 | 311.62 | 327.26 |
| IFN-γ | 510.57 | 419.77 | 525.015 | 477.465 | 388.63 | **558.45*** |
| IP-10 | **15122.21****** | 3791.48 | 14413.73 | 16653.38 | 14344.92 | 18100.87 |
| MCP-1 | 1406.6 | **1842.45*** | 1236.975 | 1519.73 | 1176.175 | **1495.29*** |
| MIP-1α | 693.39 | 559.55 | 767.89 | 578.04 | 751.31 | 689.46 |
| PDGF-bb | 3437.1 | 3134.44 | 3254.94 | 3592.29 | 3044.72 | 3594.85 |
| MIP-1β | 5173.37 | 5041.3 | 6082.05 | 5118.68 | 4584.975 | 5388 |
| RANTES | 2778.685 | 3715.47 | 2759.345 | 2778.685 | 3081.67 | 2663.27 |
| TNF-α | 640.555 | 647.3 | 687.765 | 603.06 | 586.655 | 768.705 |
| VEGF | 174.975 | 178.27 | 169.275 | 175.545 | 180.405 | 166.43 |

^a^ Data is presented as median. Statistical difference between the different sub-groups indicated by the columns was determined using a Mann-Whitney *U* test or an unpaired t-test. Statistically significant values are highlighted in bold.

**Supplementary Figure 1.**


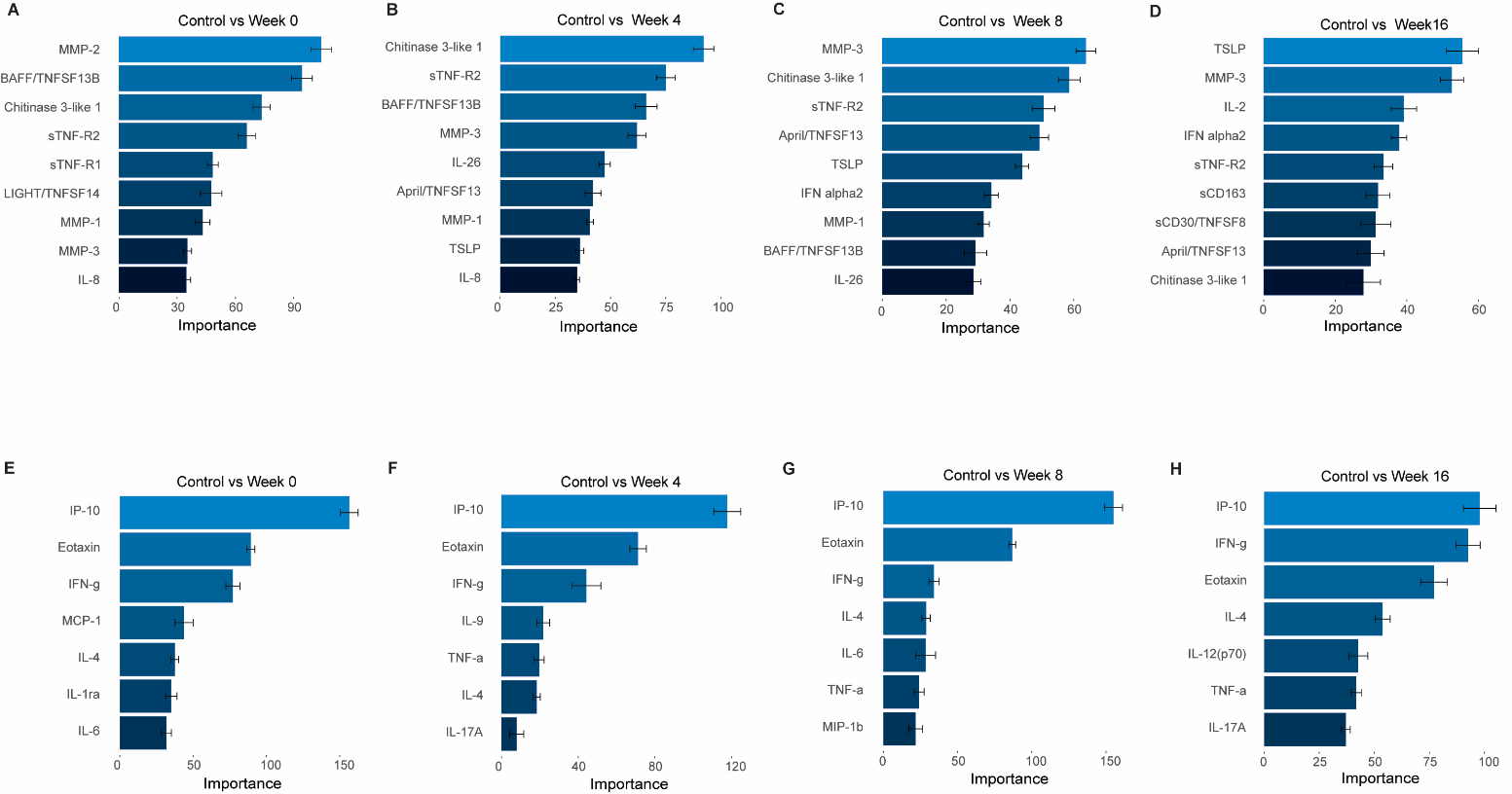


Importance ranking and dimensionality reduction facilitate cluster generation of multiplex data from longitudinal samples obtained from TB patients and healthy controls. Random forest (RF) analyses of acquired 37-plex inflammation data comparing the healthy controls with TB patients at **(A)** week 0, **(B)** week 4, **(C)** week 8, **(D)** and week 16. Corresponding RF analyses of acquired 27-plex cytokine data comparing the healthy controls with TB patients at **(E)** week 0, **(F)** week 4, **(G)** week 8, **(H)** and week 16.

**
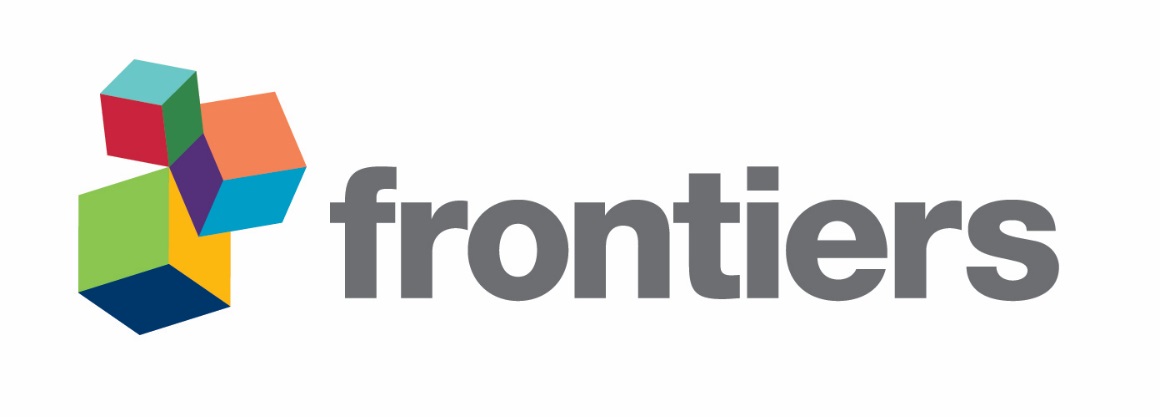
**
